# Understanding the age spectrum of respiratory syncytial virus associated hospitalisation and mortality burden based on statistical modelling methods: a systematic analysis

**DOI:** 10.1186/s12916-023-02932-5

**Published:** 2023-06-26

**Authors:** Bingbing Cong, Izzie Dighero, Tiantian Zhang, Alexandria Chung, Harish Nair, You Li

**Affiliations:** 1 Department of Epidemiology, School of Public Health, Nanjing Medical University, Nanjing, 211166 China; 2 Centre for Global Health, Usher Institute, University of Edinburgh, Edinburgh, UK; 3 MRC/Wits Rural Public Health and Health Transitions Research Unit (Agincourt), School of Public Health, Faculty of Health Sciences, University of the Witwatersrand, Johannesburg, South Africa

**Keywords:** Respiratory syncytial virus, Model, Hospitalisation, Mortality, Burden of disease, Systematic reviews

## Abstract

**Background:**

Statistical modelling studies based on excess morbidity and mortality are important for understanding RSV disease burden for age groups that are less frequently tested for RSV. We aimed to understand the full age spectrum of RSV morbidity and mortality burden based on statistical modelling studies, as well as the value of modelling studies in RSV disease burden estimation.

**Methods:**

The databases Medline, Embase and Global Health were searched to identify studies published between January 1, 1995, and December 31, 2021, reporting RSV-associated excess hospitalisation or mortality rates of any case definitions using a modelling approach. All reported rates were summarised using median, IQR (Interquartile range) and range by age group, outcome and country income group; where applicable, a random-effects meta-analysis was conducted to combine the reported rates. We further estimated the proportion of RSV hospitalisations that could be captured in clinical databases.

**Results:**

A total of 32 studies were included, with 26 studies from high-income countries. RSV-associated hospitalisation and mortality rates both showed a U-shape age pattern. Lowest and highest RSV acute respiratory infection (ARI) hospitalisation rates were found in 5–17 years (median: 1.6/100,000 population, IQR: 1.3–18.5) and < 1 year (2235.7/100,000 population, 1779.1–3552.5), respectively. Lowest and highest RSV mortality rates were found in 18–49 years (0.1/100,000 population, 0.06–0.2) and ≥ 75 years (80.0/100,000 population, 70.0–90.0) for high-income countries, respectively, and in 18–49 years (0.3/100,000 population, 0.1–2.4) and < 1 year (143.4/100,000 population, 143.4–143.4) for upper-middle income countries. More than 70% of RSV hospitalisations in children < 5 years could be captured in clinical databases whereas less than 10% of RSV hospitalisations could be captured in adults, especially for adults ≥ 50 years. Using pneumonia and influenza (P&I) mortality could potentially capture half of all RSV mortality in older adults but only 10–30% of RSV mortality in children.

**Conclusions:**

Our study provides insights into the age spectrum of RSV hospitalisation and mortality. RSV disease burden using laboratory records alone could be substantially severely underreported for age groups ≥ 5 years. Our findings confirm infants and older adults should be prioritised for RSV immunisation programmes.

**Trial registration:**

PROSPERO CRD42020173430.

**Supplementary Information:**

The online version contains supplementary material available at 10.1186/s12916-023-02932-5.

## Background

Respiratory syncytial virus (RSV) is a leading cause of acute lower respiratory infection (ALRI) in young children and older adults and has caused substantial morbidity and mortality burden, especially in low- and middle-income countries (LMICs) [1–3]. Several RSV prophylactic products targeting infants and older adults are in late-phase development and have shown promise in administration to the general population [4–7]. It is therefore important to understand the RSV disease burden across the full age spectrum in the context of prioritising age groups for immunisation programmes.

However, it is widely acknowledged that there are substantial variations in testing practice for RSV diagnosis across different ages, with young children more likely to be tested for RSV than adults, posing challenges to the estimation of RSV disease burden in adults due to under-testing [8]. This is further complicated by the imperfect sensitivity of testing methods such as serological test and rapid antigen tests. Moreover, RSV is not always documented as a main cause for hospitalisation among adults especially when they have underlying medical conditions, leading to under-reporting in medical records and because of hospitalisations and deaths associated with RSV usually occurring some days after the initial infection and atypical or delayed clinical symptoms with lower viral loads in adults, by which time the virus may no longer be detectable [9, 10]. To overcome these challenges, statistical modelling methods were developed as an alternative approach to account for under-testing and under-reporting by estimating the excess disease burden relative to a defined baseline disease burden outside RSV seasons, at a population level. Although there have been a number of statistical modelling studies in recent years that estimated RSV disease burden in multiple age groups, there are no existing reviews on their methodologies or the estimates reported therein.

In this study, we conducted a systematic review of statistical modelling studies reporting RSV hospitalisation and mortality burden to synthesise estimates on RSV hospitalisation and mortality burden across all ages and to critically appraise their methodologies. We further quantified the proportion of RSV disease burden that could be captured by medical records alone.

## Methods

### Search strategy and selection criteria

This systematic review was reported according to the Preferred Reporting Items for Systematic Reviews and Meta-Analyses (PRISMA) guidelines (see Additional file 1: Text S1) and synthesis without meta-analysis in systematic reviews (SWiM) reporting guideline (see Additional file 1: Text S2) [11, 12]. The protocol of this review was registered in the International Prospective Register of Systematic Review (PROSPERO) with registration number CRD42020173430. We searched 3 electronic databases (Medline, Embase and Global Health) for studies published between 1 January 1995 and 31 December 2021 that reported RSV-associated hospitalisation and mortality estimates based on statistical modelling methods using a tailored search strategy (see Additional file 1: Text S3). The search was limited to primary literature published in English language. References cited in retrieved articles were also examined for eligibility. The following selection criteria were applied.

#### Inclusion criteria

- Reporting data for RSV-infected humans; AND
- Reporting rates for RSV mortality and/or hospitalisation based on statistical modelling rather than direct observation; AND
- Reporting estimates on rates for RSV mortality and/or hospitalisation for any age groups.

#### Exclusion criteria

- Investigating RSV as a co-infection rather than a primary outcome; OR
- Case definition not clearly defined or inconsistently applied; OR
- Case-reports, systematic reviews, narrative reviews, letters to editors, or commentary; OR
- Focusing on subjects with special medical conditions (e.g. preterm and HIV-infected).

### Systematic literature review

Two groups of reviewers (BC and ID; TZ and AC) independently screened titles, abstracts and full-texts of the retrieved records from the literature search and extracted data using a tailored data extraction template. The data extraction template consisted of two parts: the first part collected study-level characteristics such as the study location, time period, age groups, statistical method, study model characteristics and case definition; the second part collected data on outcome measures (i.e. incidence rates and 95% confidence intervals) for each age group and case definition. Any discrepancies during data screening and extraction were resolved within the review team. A sample form of data extraction is available in Additional file 1: Text S4.

### Quality assessment

We developed a modified quality assessment checklist based on the PROBAST risk of bias assessment tool for model studies [13]. The questionnaire contained six questions and answer to each of the questions could be “yes”, “no”, or “unclear”, corresponding to 1, 0 and 0 point, respectively. We calculated the overall score for each study after assessing each criterion as listed above; studies were defined as “high quality” (6 points), “moderate quality” (4–5 points) and “low quality” (≤ 3 points). Details are shown in Additional file 1: Table S1. Any discrepancies during quality assessment were resolved within the review team.

### Data analysis

Given that not all studies reported confidence intervals of the estimates (i.e. only point estimates were provided), we first calculated the median, interquartile range (IQR) and range of the reported point estimates stratified by age group, case definition and World Bank country income group. The following age groups were used: < 1 year, 1–4 years, 5–17 years, 18–49 years, 50–64 years, 65–74 years, ≥ 75 years and 0–4 years, ≥ 65 years (if available). When the age group of reporting did not match exactly with the defined age groups above, the nearest defined age group was used for extraction. Data recorded during the 2009 influenza pandemic were excluded to avoid bias associated with the changes in the inter-seasonal baseline estimates as a result of the pandemic. We conducted a sensitivity analysis that excluded studies with high risks of bias (defined as quality scores ≤ 3).

When studies reported both estimates from statistical modelling methods and directly from medical records, these estimates were compared by age group and case definition to help quantify the potential under-testing and under-reporting of RSV hospitalisation and mortality burden. For studies reporting RSV-associated hospitalisation or mortality in terms of multiple case definitions, we calculated median (IQR) of ratio of modelled estimates in different case definitions by age group. When studies reported estimates for multiple years or seasons, year-on-year variability was visually evaluated by a line graph. When a study used different modelling approaches for the same estimation (e.g. a rate difference method with two different baseline periods), we considered only the estimate from the main analysis defined by the study authors.

When at least two studies that reported both the point estimate and confidence interval of estimates for a given age group, case definition and World Bank country income group, we performed meta-analysis using a multilevel model with random effects at the study level: level 1 accounted for within-study variations that could also include year-on-year variations if estimates from individual studies were reported on a year-by-year basis; level 2 accounted for between-study variations with random effects [14, 15]. The detailed information of the model is presented in Additional file 1: Text S5. The choice of random effects at the study level was based on the anticipation that the included studies could differ by region, study period, population characteristics, diagnostic criteria and modelling methods. We used meta-analysis as a sensitivity analysis as some studies did not report the statistical uncertainty range necessary for carrying out meta-analysis.

All statistical analyses and data visualisations were performed with R version 4.2.1.

## Results

### Study characteristics

**Fig. 1:**
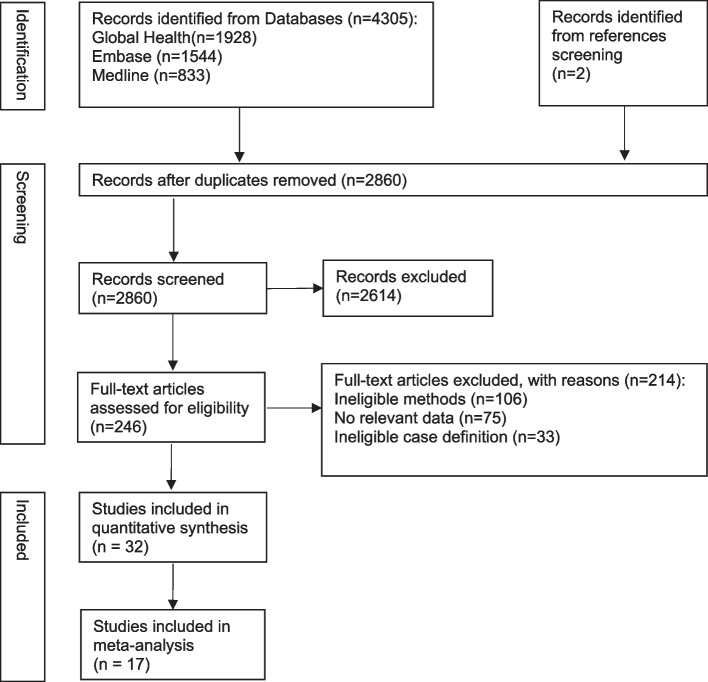
PRISMA diagram for selection of studies

A total of 2860 records were identified for title and abstract screening (after exclusion of duplicates), of which 246 records were further assessed by full-text. Finally, a total of 32 studies were included, with 17 studies included in meta-analysis (Fig. 1).

Overall, 19 studies reported RSV-associated hospitalisation rate. Of these, 14 studies [10, 16–19, 19–27] reported using a case definition of acute respiratory infection (ARI) or broad respiratory hospitalisation. Seventeen studies reported mortality estimates, of which 13 studies [23, 25, 28–38] reported all-cause mortality. Four studies [10, 19, 35, 38] reported both hospitalisation and mortality estimates. The majority of studies (26/32) were from high-income countries. Geographical distribution of studies included is presented in Additional file 1: Figure S1. Age groups and case definitions varied substantially across the included studies. The basic characteristics of the included studies are available in Additional file 1: Tables S2 and S3.

Regarding methodology, 27 studies used regression models with an RSV activity proxy. All of them used polynomial or trigonometric functions of time to control for temporal trends and/or seasonality in hospitalisation or mortality. The majority of the generalised linear models assumed that hospitalisation or mortality followed a normal distribution with an identity link or a Poisson distribution with a log link. Six studies used rate-difference models (Additional file 1: Table S4) and no studies used Serfling-type methods [39]. Time lag between RSV activity and outcome, climatic factors (i.e. temperature or humidity) and influenza activity were the covariates most commonly included in the regression models. We found eight studies applying time lags selected through model fit comparison; 2, 4 and 2 studies used time lags of 2, 3 and 4 weeks, respectively. Model characteristics are summarised in Table 1 and further details are provided in Additional file 1: Text S6 and Text S7. Reported average annual estimates of RSV associated hospitalisation or mortality by studies with various modelling methods listed are shown in Additional file 1: Tables S5-10. Details of modelling and non-modelling approaches for estimation of RSV disease burden are given in Additional file 1: Tables S11 and S12. Overall, 3, 18 and 11 studies were assessed as “high quality”, “moderate quality” and “low quality”, respectively.

**Table 1:** Summary of characteristics of modelling techniques used in included studies

Characteristics	No. of studies (*n* = 32) ^a^	Reference(s)
Statistical modelling technique		
Regression model with a proxy for RSV activity	27	[10, 16–28, 30–34, 36–38, 40–44]
Rate-difference	6	[29, 34, 35, 45–47]
Bayesian inference	1	[18]
Serfling-type	0	__
Time lag between RSV activity and outcome in regression models		
No	19	[16, 17, 19–25, 28, 31–34, 36, 40–43]
Yes	8	[10, 18, 26, 27, 30, 37, 38, 44]
Climatic covariates included in regression models		
No	14	[10, 17, 19–21, 26–28, 38, 40–42, 44, 45]
Temperature	13	[16, 18, 22–25, 30, 32–34, 36, 37, 43]
Humidity	2	[16, 18]
RSV activity ‘proxy’ used in regression models		
Number of RSV-positive laboratory samples	17	[14, 19–25, 27–32, 40, 42, 44]
Proportion of RSV-positive laboratory samples	7	[22, 27, 34, 36, 42–44]
RSV-coded ALRI admission	3	[23–25]
RSV-coded death certificate	1	[31]
Whether other pathogen activity was considered in the regression models		
Influenza	13	[10, 17, 19, 22, 27, 28, 30, 33, 36, 40, 41, 43, 44]
Influenza and others	14	[16, 18, 20, 21, 23–26, 31, 32, 34, 37, 38, 42]

*RSV *respiratory syncytial virus

^a^Multiple statistical methods or models could have been used in a single study to estimate RSV-associated hospitalisation or mortality; therefore, different methods/models were counted separately and only statistical methods used in the main analysis of each study were included in our analysis. Therefore, the subtotal in each category may differ from the total number of studies (*n* = 32). For example, reference 34 used both rate-difference and regression model

### Hospitalisation rates

**Fig. 2:**
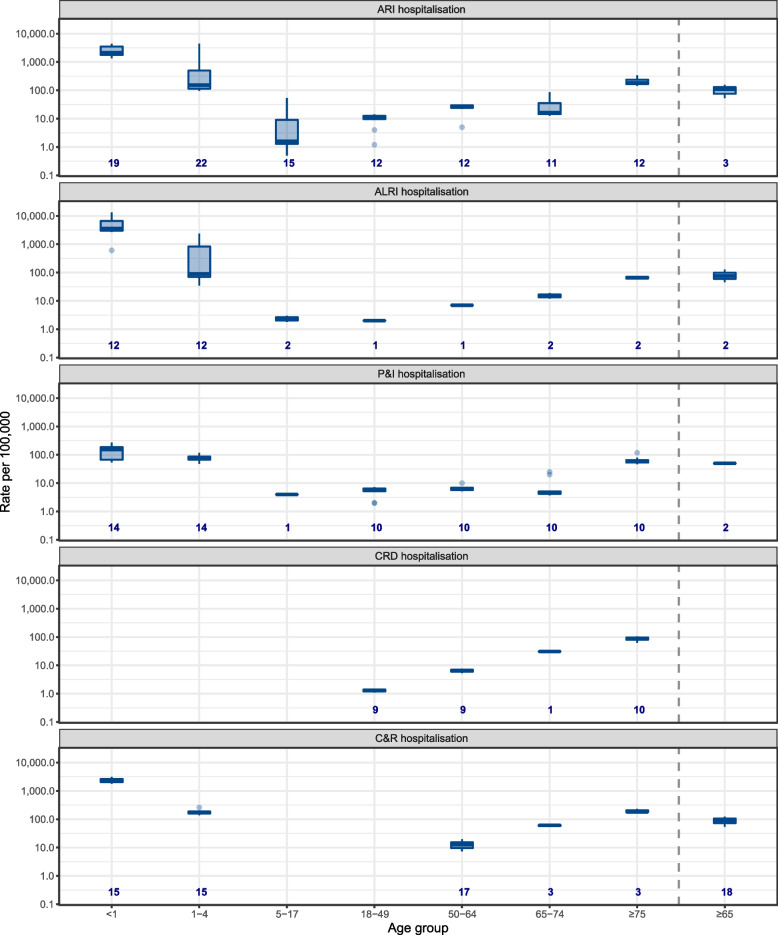
Median, IQR and ranges of reported estimates of rates of RSV-associated hospitalisation in high-income countries. ALRI, acute lower respiratory infection; ARI, acute respiratory infection; C&R, circulatory and respiratory; CRD, chronic respiratory infection; P&I, pneumonia and influenza. *Y*-axis is displayed in log scale. The dotted line is used for distinguishing the estimates for broader age groups from finer age groups. Number at the bottom of each panel indicates number of data-points, if studies reported estimates for multiple years or seasons, then this study contributes to multiple data points. This data is available in Additional file 1: Tables S5-S6, S8-10

For high-income countries, the highest rate of RSV hospitalisation was found in infants < 1 year old across all case definitions. RSV hospitalisation rates showed a U-shape age pattern (Fig. 2). Rates of RSV hospitalisation among infants < 1 year old ranged from 1817.2 to 3150.7 per 100,000 (general) population, with a median of 2227.7 (IQR, 2029.5–2640.4) for circulatory and respiratory (C&R) diseases; 1353 to 7601 per 100,000 population with a median of 2235.7 (IQR, 1779.1–3552.5) for ARI; 608.2 to 13,317.2 per 100,000 population with a median of 3560.0 (IQR, 3012.5–6626.1) for ALRI; and 54 to 3055 per 100,000 population with a median of 157.5 (IQR, 68.5–199.5) for pneumonia and Influenza (P&I). The lowest rate of RSV hospitalisation was found in 18–49 years for P&I and ALRI and in 5–17 years for ARI. More detailed results are in Additional file 1: Fig. 2 and Table S13. Sensitivity analyses that excluded low-quality studies with high risk of bias showed similar median estimates (Additional file 1: Table S14).

For the three age groups with available estimates from both high-income and upper-middle-income countries, < 1 year, 1–4 years and 5–17 years, RSV hospitalisation rates were consistently higher in upper-middle-income countries than high-income countries within the same case definition, with rate ratios ranging from 3.6 to 35.6 (Additional file 1: Figure S2).

**Fig. 3:**
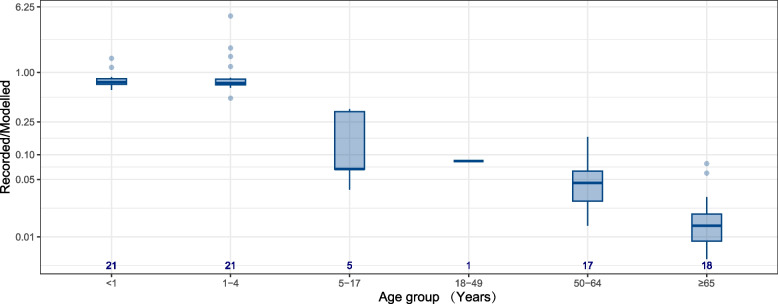
Comparison of modelled and recorded estimates by age group for studies reporting RSV-associated hospitalisation rates. The *y*-axis represents the ratio of recorded estimates to modelled estimates and is displayed in log scale. Number at the bottom of indicates number of data-points, if studies reported estimates for multiple years or seasons, then this study contributes to multiple data points. Recorded estimates, also called directly observed number/rate, represents hospital admissions with laboratory confirmed virus infection. Modelled estimates represent hospital admissions rate estimated from statistical models

A clear decreasing trend in the ratio of recorded and modelled RSV hospitalisation rates was observed with increasing age (studies included for this analysis were all rated as moderate and high quality). While recorded RSV hospitalisation burden in < 1 year and 1–4 years could account for 70% of modelled burden, the proportion reduced to less than 10% for all age subgroups beyond 5 years (Fig. 3).

### Mortality rates

**Fig. 4:**
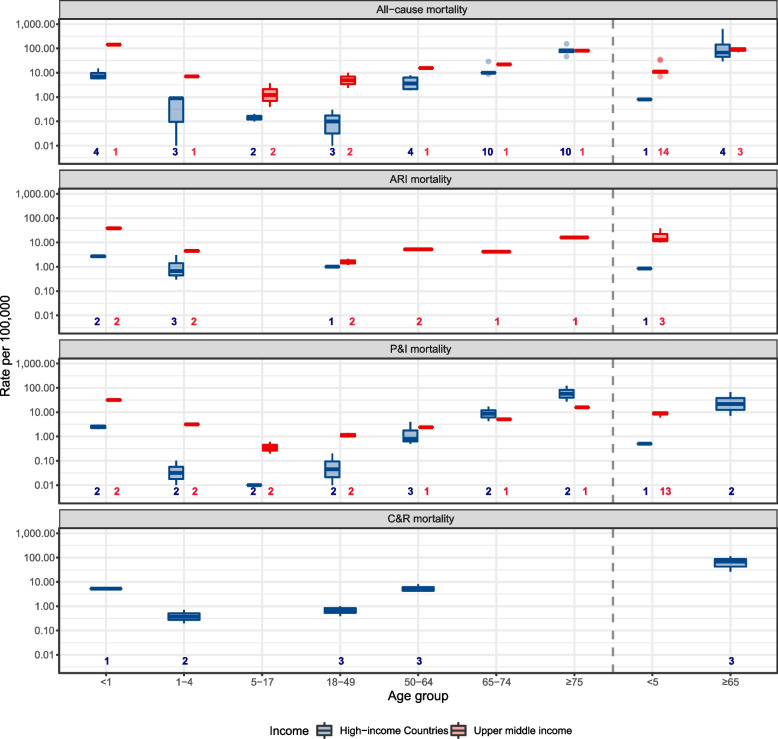
Median, IQR and ranges of reported estimates of rates of RSV-associated mortality. ARI, acute respiratory infection; C&R, circulatory and respiratory; P&I, pneumonia and influenza. *Y*-axis is displayed in log scale. The dotted line is used for distinguishing the estimates for broader age groups from finer age groups. Number at the bottom of each panel indicates number of data-points, if studies reported estimates for multiple years or seasons, then this study contributes to multiple data points. This data is available in Additional file 1: Tables S4-5, S7-9, 11–13

RSV mortality rates showed a U-shape age pattern, similar to RSV hospitalisation rates (Fig. 4). For high-income countries, the highest mortality rate was found in age group ≥ 65 years, especially for those aged 75 years and older, followed by infants < 1 year. RSV-associated all-cause and P&I mortality for those aged ≥ 65 years ranged from 29.6 to 622.0 per 100,000 population, with a median of 70.1 (IQR, 46.5–221.5) and 7.2 to 66.0 per 100,000 population with a median of 36.6 (IQR, 21.9–51.3), respectively. The lowest mortality rate was observed in age groups of 5–17 and 18–49 years, with a median of 0.1 per 100,000 population for all-cause mortality and 0.01 per 100,000 population for P&I mortality. However, for upper-middle-income countries, the highest mortality rate was found in infants < 1 year, followed by older adults aged ≥ 65 years. The median RSV mortality rate ratio between upper-middle-income countries and high-income countries was 11.0 (IQR, 2.1–16.5) across all case definitions and age groups. Interestingly, RSV-associated P&I mortality among older adults ≥ 65 years in upper-middle-income countries was lower than that in high-income countries, with a ratio of 0.48 and 0.21 for age groups of 65–74 years and ≥ 75 years, respectively (see Fig. 4 and Additional file 1: Tables S15-17). Similar results were observed in sensitivity analysis excluding low-quality studies (Additional file 1: Table S18); the only exception was for ≥ 65 years on all-cause mortality rate in high-income countries (70.1 [46.5–211.5], per 100,000 population in main analysis based on four studies and 337.1 [194.6–479.5] in sensitivity analysis based on two studies).

### Case definitions

Using all-cause mortality as the reference, P&I mortality could capture about half of the RSV-related mortality in older adults aged ≥ 65 years but only 11% to 34% in other age groups; similar findings were observed for P&I hospitalisation (using ARI hospitalisation as the reference). By comparison, C&R mortality could capture the most RSV mortality (Table 2).

**Table 2:** Comparison of median of ratio of modelled estimates in different case definitions by age group

Age groups	P&I vs all-cause mortality		ARI vs all-cause mortality		C&R vs all-cause mortality		P&I vs ARI hospitalisation		ALRI vs ARI hospitalisation	
	Ratio	No.	Ratio	No.	Ratio	No.	Ratio	No.	Ratio	No.
** < 1 y**	0.33	3	0.32	2	0.98	1	0.06	4	0.35	2
**1–4 y**	0.11	3	0.52	3	0.81	1	0.40	4	0.34	2
**5–17 y**	0.16	3	0.33	4	__	__	0.25	2	__	__
**18–49 y**	0.34	4	0.50	3	__	__	0.51	2	0.50	1
**50–64 y**	0.26	4	0.29	1	0.60	1	0.23	2	0.23	1
**65–74 y**	0.49	3	0.19	1	__	__	0.29	3	0.19	2
** ≥ 75 y**	0.59	3	0.20	1	__	__	0.34	3	0.28	2
** ≥ 65 y**	0.50	2	__	__	0.90	1	0.31	1	__	__

*y *years, *No *the number of data-points calculated median, range and IQR

If studies reported estimates for multiple years or seasons, then this study contribute to multiple data points. *ALRI*, acute lower respiratory infection; *ARI*, acute respiratory infection; *C&R*, circulatory and respiratory; *P&I*, pneumonia and influenza. The horizontal line in the table indicates that there is no relevant data

### Year-on-year variations

The median ratios of maximum to minimum of RSV-ARI hospitalisation and mortality rates were 2.2 (IQR, 1.4–4.9) and 1.7 (IQR, 1.4–2.0) across all age groups, respectively. The greatest difference between maximum and minimum rates were 8.7 and 16.8 for RSV hospitalisation and mortality, respectively, due to the effect of two outlier seasons [20, 46]. Despite the variations, no clear yearly trends in RSV-ARI hospitalisation rates or RSV mortality were observed (Additional file 1: Figures S3 and S4).

### Comparison of median and meta-analysis estimates

Forest plots of meta-analysis of age-specific RSV-associated hospitalisation and mortality rates in countries with two income levels sub-grouped by age and case definitions are shown in Additional file 1: Figures S5 and S6, respectively. The correlation between the median estimates and meta estimates of RSV-associated hospitalisation and mortality rate by case definition, age group and country income levels was high (Pearson *r* = 0.95, *P* < 0.001; Pearson *r* = 0.81, *P* < 0.001, respectively) (Additional file 1: Figure S7).

## Discussion

To the best of our knowledge, our study is the first to systematically review statistical modelling studies of RSV burden using a variety of principal hospitalisation and mortality diagnoses across the age spectrum. As expected, we found RSV-associated hospitalisation and mortality rates followed a U-shaped age pattern. Specifically, the lowest hospitalisation and mortality rates were found in age groups of 5–17 and 18–49 years. The highest rate of RSV-associated hospitalisation was found in age group < 1 year old; the highest mortality rate occurred in older adults aged ≥ 65 years in high-income countries and in infants in upper-middle-income countries. Whilst medical records capture the majority of RSV hospitalisations for children under 5 years, they would only account for less than 10% of the estimated RSV hospitalisation burden in all age subgroups aged > 5 years, especially for adults ≥ 50 years. C&R mortality would capture the most RSV mortality in different age groups. Furthermore, the case definition of P&I captures more RSV hospitalisations and mortality for older adults than young children.

Our estimates of RSV hospitalisation rate in infants and children aged 1–4 years (2236 and 171 per 100,000 population, respectively) from high-income countries were in line with a previous report by Li et al. (2200 and 160 per 100,000 population, respectively) that accounted for under-testing by applying multipliers [2]. Moreover, our RSV-ARI hospitalisation rate in infants from high-income countries was nearly identical to the median rate (2370 per 100,000 population) reported based on modelling methods in a recent systematic review focusing on US infants [48]. The estimates for RSV-ARI hospitalisation rate in those aged 18–64 years were generally lower than the range of previous estimates from two prospective cohort studies in the USA [49, 50]. This may possibly be due to systematic differences in the study populations, two cohort studies having a smaller sample size and fewer seasons compared to the modelling studies included. The RSV-ARI hospitalisation rate in older adults aged > 65 years in our study (108 per 100,000 population) was comparable with that in high-income countries from a recent updated review (145 per 100,000 population) [51]. These overall similarities suggest that modelling studies based on excess disease burden could produce robust estimates of RSV-associated disease burden. Within the age category of ≥ 65 years, RSV-ARI hospitalisation rates have been skewed heavily toward the older age groups (i.e. ≥ 75 years old) — the hospitalisation rates in those aged ≥ 75 years was 10 times higher than that of those aged 65–74 years, which is consistent with a recent multi-country study [52]. In addition, our findings suggest that RSV-associated hospitalisations with chronic respiratory disease (CRD) diagnosis among older adults accounted for approximately half of RSV-associated ARI hospitalisations. This indicates that older adults with CRD represent a substantial proportion of RSV-associated hospitalisation burden and should be considered as a priority group for RSV immunisation programmes.

Our study showed that the difference between directly recorded and modelled hospitalisation rates varied considerably among different age groups. This suggests that RSV hospitalisation rates for patients aged ≥ 5 years old that are based on databases with laboratory confirmed virus infections may be substantially underestimated; this was supported by a study reporting that the completeness of RSV identification were 66% and 9% for paediatric and adult population, respectively [25]. This highlights the value of modelling studies for understanding the true disease burden among adolescents and adults. McLaughlin and colleagues compared RSV hospitalisation rates in infants [48] and adults [53] in the USA estimated from different sources, including active surveillance, retrospective medical records review, claim databases (e.g. mainly based on ICD codes), and modelling studies. They found that for infants, modelling studies generated the highest estimates, followed by claim database and retrospective medical records review, and active surveillance generated the lowest estimates; for adults, however, active surveillance generated a higher estimate than modelling studies. This suggests that further prospective cohort study with active case ascertainment is needed to more accurately evaluate the hospitalisation burden associated with RSV infections in adults.

RSV mortality burden forms an important component of the overall RSV disease burden and is fundamental in assessing cost-effectiveness of interventions. However, mortality attributed to RSV may be underestimated as deaths can occur outside of health facilities without reliable documentation regarding cause of death, and even for in-hospital mortality, it is often challenging to determine the causal role of RSV in mortality not only because RSV testing is not routinely conducted but also because of the presence of underlying medical conditions which can contribute in varying degrees to a patient’s death. Statistical modelling studies based on the statistical correlations between RSV and mortality offer an alternative approach to indirectly assessing RSV mortality burden. We compared the RSV mortality estimates in our study with the estimates of RSV-associated all-cause mortality in children under five years from a global systematic analysis [2]; we found that estimates for age group < 1 year, 1–4 years and < 5 years in high-income countries were comparable between the two studies (1829 vs 1900, 361 vs 400 and 2190 vs 2300 per 100,000 population, respectively).

Regarding case definitions used in the modelling studies, we showed that P&I mortality could capture half of all RSV mortality in older adults but only 10–30% of RSV mortality in children, whereas it was mainly based on low-quality evidence (3/4). Similar findings were observed for P&I hospitalisation. The varying sensitivity of the P&I case definition in different age groups has implications for the conduct and interpretation of studies that reanalysed clinical databases.

In terms of methodology, regression modelling methods were the most commonly used approach for estimating RSV-associated excess disease burden. This method utilised time-series data of outcome and covariates including an RSV activity proxy and was also able to incorporate time lags and other potential confounders as covariates (e.g. co-circulating pathogens and meteorological factors). The use of time lags was useful in accounting for the time difference between initial infection and hospitalisation/death. However, regression modelling methods require data which have been collected over a longer period of time (e.g. at least five years) and stable time series of both the outcome and covariates, which might not be available in resource-poor settings. An alternative approach that has been commonly used is the rate-difference method, which is based on the difference in disease burden between a predefined RSV season and a baseline period. However, compared with regression modelling methods, it is more challenging for rate-difference methods to account for the impact of other co-circulating pathogens or metrological factors. To account for potential heterogeneities in methodology based on the factors above discussed, we conducted sensitivity analysis excluding studies with high risks of bias and showed broadly similar results to the main analyses.

Our results should be interpreted with caution in the context of several limitations. First, we could not fully account for heterogeneities across individual studies due to the underlying variation in case definitions, study population and study period. Second, some studies reported negative estimates for the lower bound of the statistical uncertainty range, likely a result of insufficient statistical power (e.g. short study period) or suboptimal modelling framework (e.g. not accounting for other co-circulating pathogens) and a number of studies did not report the statistical uncertainty range for the estimates. As a result, we had to exclude these studies from the meta-analysis and used median estimates as the main estimate for reporting (although sensitivity analysis comparing estimates from meta-analysis and median showed no substantial differences). Third, few modelling studies reported finer age bands with the first year of life, which is more relevant to RSV immunisation strategies. A recently published modelling study across 6 European countries showed that annual RSV-associated hospitalisation rate was as high as over 40 per 1000 population in the age group of 0–2 months [52]. Fourth, the vast majority of modelling studies included in this analysis were conducted in high-income countries; low- and middle-income countries, especially low- and lower-middle income countries, were underrepresented in this analysis. Fifth, few modelling studies included age-specific RSV activity data in the model (e.g. they used all-age RSV activity instead) for estimating disease burden in certain age groups; if the timing of RSV season varied by age, then this could bias the age-specific estimate. Finally, all included studies reported estimates only for the pre-COVID-19 pandemic period; recent studies highlighted the drastic changes in the circulating timing and attack rate as well as severity of RSV infections after the onset of the COVID-19 pandemic [54–56]. Therefore, the hospitalisation and mortality rates from our study should be interpreted as the inter-pandemic “normality”, although it is still unknown whether RSV epidemiology would return to the pre-pandemic “normality”. Nonetheless, we argue that the age profile of RSV hospitalisation and mortality rates reported in our study should not change dramatically during the pandemic. This is supported by recent studies from France and Denmark reporting that the age distribution of medically attended RSV episodes was generally similar between pre-epidemic and pandemic periods [56, 57].

## Conclusions

Despite these limitations, by synthesising all available data from the literature, our study provides a comprehensive review of the existing evidence based on statistical modelling approaches regarding RSV-associated mortality and hospitalisation burden of the full age spectrum. Our findings help confirm on the subpopulations of priority for planning of future RSV intervention programmes.

## Supplementary Information

**Additional file 1:** Additional details on methods, characteristics of included studies, quality assessment, results of sensitivity and meta-analysis. **Text S1.** PRISMA 2020 Checklist. **Text S2.** Synthesis without meta-analysis in systematic reviewsreporting guideline. **Text S3.** Search Strategies. **Text S4.** Data extraction proforma. **Text S5.** Description of multilevel model with random effects. Text S6. Modelling methods. **Text S7.** Model characteristics. **Figure S1.** Geographical distribution of studies included. **Figure S2.** Median, IQR and ranges of reported estimates of rates of RSV-associated hospitalisation by country income level. **Figure S3.** Annual rates of RSV-associated ARI hospitalisation per 100,000 over time by study and age group. **Figure S4.** Annual rates of RSV-associated mortality per 100,000 over time by study and age group. **Figure S5.** Forest plots of meta-analysis of age-specific RSV-associated hospitalisation rates in high income countries by age group and case definition. **Figure S6.** Forest plots of meta-analysis of age-specific RSV-associated mortality rates in upper-middle-income countries by age group and case definition. **Figure S7.** Comparison of median estimatesand meta estimatesfor RSV-associated hospitalisation and mortality. **Table S1.** Risk of bias and quality assessment for included studies. **Table S2.** Summary of modelling studies reporting RSV-associated hospitalisation. **Table S3.** Summary of modelling studies reporting RSV-associated mortality. **Table S4.** Definitions of RSV-epidemic periods used in studies applying rate-difference methods. **Table S5.** Summary of the reported average annual estimates of RSV all-cause mortality rate. **Table S6.** Summary of the reported average annual estimates of RSV-C&R hospitalisation and mortality rate. **Table S7.** Summary of the reported average annual estimates of RSV-ARI hospitalization rate. **Table S8.** Summary of the reported average annual estimates of RSV-ARI mortality rate. **Table S9.** Summary of the reported average annual estimates of RSV-ALRI hospitalisation and mortality rate. **Table S10.** Summary of the reported average annual estimates of RSV-P&I hospitalisation and mortality rate. **Table S11.** Methods for estimating RSV-associated hospitalisation and mortality. **Table S12.** Modelling approaches for estimation of RSV-associated hospitalisation and mortality. **Table S13.** Median and range of reported estimates of ALRI, P&I, C&R and ARI hospitalisation associated with RSV per 100,000 by age group in high income countries. **Table S14.** Median and range of reported estimates of ALRI, P&I, C&R and ARI hospitalisation associated with RSV per 100,000 by age group in high income countries when excluding low-quality studies. **Table S15.** Median, IQR and range of reported estimates of all-cause, C&R, ARI and P&I mortality associated with RSV per 100,000 by age group globally. **Table S16.** Median, IQR and range of reported estimates of all-cause, C&R, ARI and P&I mortality associated with RSV per 100,000 by age group in high income countries. **Table S17.** Median, IQR and range of reported estimates of all-cause, C&R, ARI and P&I mortality associated with RSV per 100,000 by age group in upper-middle-income countries. **Table S18.** Median, IQR and range of reported estimates of all-cause, C&R, ARI and P&I mortality associated with RSV per 100,000 by age group in high income countries when excluding low-quality studies.

## Data Availability

The datasets supporting the conclusions of this article and corresponding R codes can be found in the GitHub repository: https://github.com/cbb666/RSV_modelling_work.

## Abbreviations

ALRI: Acute lower respiratory infection
ARI: Acute respiratory infection
C&R: Circulatory and respiratory
COVID-19: The coronavirus disease 2019
IQR: Interquartile range
LMICs: Low- and middle-income countries
P&I: Pneumonia and influenza
PRISMA: The Preferred Reporting Items for Systematic Reviews and Meta-Analyses guidelines
RSV: Respiratory syncytial virus
SWiM: Synthesis without meta-analysis in systematic reviews reporting guideline
