# Supplementary material for: Understanding the age spectrum of respiratory syncytial virus associated hospitalisation and mortality burden based on statistical modelling methods: a systematic analysis
Source: BMC Med. 2023 Jun 26;21:224. doi: 10.1186/s12916-023-02932-5 (PMC10294405; doi:10.1186/s12916-023-02932-5)
Supplement: Supplementary file 1 — Additional file 1: Additional details on methods, characteristics of included studies, quality assessment, results of sensitivity and meta-analysis. Text S1. PRISMA 2020 Checklist. Text S2. Synthesis without meta-analysis in systematic reviewsreporting guideline. Text S3. Search Strategies. Text S4. Data extraction proforma. Text S5. Description of multilevel model with random effects. Text S6. Modelling methods. Text S7. Model characteristics. Figure S1. Geographical distribution of studies included. Figure S2. Median, IQR and ranges of reported estimates of rates of RSV-associated hospitalisation by country income level. Figure S3. Annual rates of RSV-associated ARI hospitalisation per 100,000 over time by study and age group. Figure S4. Annual rates of RSV-associated mortality per 100,000 over time by study and age group. Figure S5. Forest plots of meta-analysis of age-specific RSV-associated hospitalisation rates in high income countries by age group and case definition. Figure S6. Forest plots of meta-analysis of age-specific RSV-associated mortality rates in upper-middle-income countries by age group and case definition. Figure S7. Comparison of median estimatesand meta estimatesfor RSV-associated hospitalisation and mortality. Table S1. Risk of bias and quality assessment for included studies. Table S2. Summary of modelling studies reporting RSV-associated hospitalisation. Table S3. Summary of modelling studies reporting RSV-associated mortality. Table S4. Definitions of RSV-epidemic periods used in studies applying rate-difference methods. Table S5. Summary of the reported average annual estimates of RSV all-cause mortality rate. Table S6. Summary of the reported average annual estimates of RSV-C&R hospitalisation and mortality rate. Table S7. Summary of the reported average annual estimates of RSV-ARI hospitalization rate. Table S8. Summary of the reported average annual estimates of RSV-ARI mortality rate. Table S9. Summary of the reported average annual e [file 12916_2023_2932_MOESM1_ESM.docx]

**Supplemental Material**

**Respiratory syncytial virus associated hospitalisation and mortality of all age spectrum based on statistical modelling methods: a systematic review**

**Contents**

[Detailed description of methodology	2](#_Toc136303167)

[Text S1. PRISMA 2020 Checklist	2](#_Toc136303168)

[Text S2. Synthesis without meta-analysis in systematic reviews (SWiM) reporting guideline.	4](#_Toc136303169)

[Text S3. Search Strategies	5](#_Toc136303170)

[Text S4. Data extraction proforma	7](#_Toc136303171)

[Text S5. Description of multilevel model with random effects	7](#_Toc136303172)

[Text S6. Modelling methods	8](#_Toc136303173)

[Text S7. Model characteristics	9](#_Toc136303174)

[Supplementary figures	10](#_Toc136303175)

[Figure S1. Geographical distribution of studies included.	10](#_Toc136303176)

[Figure S2. Median, IQR and ranges of reported estimates of rates of RSV-associated hospitalisation by country income level.	10](#_Toc136303177)

[Figure S3. Annual rates of RSV-associated ARI hospitalisation per 100,000 over time by study and age group.	11](#_Toc136303178)

[Figure S4. Annual rates of RSV-associated mortality per 100,000 over time by study and age group.	12](#_Toc136303179)

[Figure S5. Forest plots of meta-analysis of age-specific RSV-associated hospitalisation rates in high income countries by age group and case definition.	13](#_Toc136303180)

[Figure S6. Forest plots of meta-analysis of age-specific RSV-associated mortality rates in upper-middle income countries by age group and case definition.	17](#_Toc136303181)

[Figure S7. Comparison of median estimates (main analysis) and meta estimates (“sensitivity analysis”) for RSV-associated hospitalisation and mortality rates.	20](#_Toc136303182)

[Supplementary tables of results	21](#_Toc136303183)

[Table S1. Risk of bias and quality assessment for included studies	21](#_Toc136303184)

[Table S2. Summary of modelling studies reporting RSV-associated hospitalisation.	23](#_Toc136303185)

[Table S3. Summary of modelling studies reporting RSV-associated mortality.	25](#_Toc136303186)

[Table S4. Definitions of RSV-epidemic periods used in studies applying rate-difference methods.	27](#_Toc136303187)

[Table S5. Summary of the reported average annual estimates of RSV all-cause mortality rate (per 100,000).	28](#_Toc136303188)

[Table S6. Summary of the reported average annual estimates of RSV-C&R hospitalisation and mortality rate (per 100,000).	29](#_Toc136303189)

[Table S7. Summary of the reported average annual estimates of RSV-ARI hospitalization rate (per 100,000).	30](#_Toc136303190)

[Table S8. Summary of the reported average annual estimates of RSV-ARI mortality rate (per 100,000)	31](#_Toc136303191)

[Table S9. Summary of the reported average annual estimates of RSV-ALRI hospitalisation and mortality rate (per 100,000)	32](#_Toc136303192)

[Table S10 Summary of the reported average annual estimates of RSV-P&I hospitalisation and mortality rate (per 100,000).	33](#_Toc136303193)

[Table S11. Methods for estimating RSV-associated hospitalisation and mortality.	34](#_Toc136303194)

[Table S12. Modelling approaches for estimation of RSV-associated hospitalisation and mortality.	35](#_Toc136303195)

[Table S13. Median and range of reported estimates of ALRI, P&I, C&R and ARI hospitalisation associated with RSV per 100,000 by age group in high income countries.	36](#_Toc136303196)

[Table S14. Median and range of reported estimates of ALRI, P&I, C&R and ARI hospitalisation associated with RSV per 100,000 by age group in high income countries when excluding low-quality studies.	37](#_Toc136303197)

[Table S15. Median, IQR and range of reported estimates of all-cause, C&R, ARI and P&I mortality associated with RSV per 100,000 by age group globally (regardless of income level).	38](#_Toc136303198)

[Table S16. Median, IQR and range of reported estimates of all-cause, C&R, ARI and P&I mortality associated with RSV per 100,000 by age group in high income countries.	39](#_Toc136303199)

[Table S17. Median, IQR and range of reported estimates of all-cause, C&R, ARI and P&I mortality associated with RSV per 100,000 by age group in upper-middle income countries.	40](#_Toc136303200)

[Table S18. Median, IQR and range of reported estimates of all-cause, C&R, ARI and P&I mortality associated with RSV per 100,000 by age group in high income countries when excluding low-quality studies.	41](#_Toc136303201)

# Detailed description of methodology

## Text S1. PRISMA 2020 Checklist

| Section and Topic | Item # | Checklist item | Location where item is reported |
| --- | --- | --- | --- |
| TITLE | | |  |
| Title | 1 | Identify the report as a systematic review. | 1 |
| ABSTRACT | | |  |
| Abstract | 2 | See the PRISMA 2020 for Abstracts checklist. | 2-3 |
| INTRODUCTION | | |  |
| Rationale | 3 | Describe the rationale for the review in the context of existing knowledge. | 3-4 |
| Objectives | 4 | Provide an explicit statement of the objective(s) or question(s) the review addresses. | 4 |
| METHODS | | |  |
| Eligibility criteria | 5 | Specify the inclusion and exclusion criteria for the review and how studies were grouped for the syntheses. | 5 |
| Information sources | 6 | Specify all databases, registers, websites, organisations, reference lists and other sources searched or consulted to identify studies. Specify the date when each source was last searched or consulted. | 4-5 |
| Search strategy | 7 | present the full search strategies for all databases, registers and websites, including any filters and limits used. | Appendix pp 6-7 |
| Selection process | 8 | Specify the methods used to decide whether a study met the inclusion criteria of the review, including how many reviewers screened each record and each report retrieved, whether they worked independently, and if applicable, details of automation tools used in the process. | 4-5 |
| Data collection process | 9 | Specify the methods used to collect data from reports, including how many reviewers collected data from each report, whether they worked independently, any processes for obtaining or confirming data from study investigators, and if applicable, details of automation tools used in the process. | 5 |
| Data items | 10a | List and define all outcomes for which data were sought. Specify whether all results that were compatible with each outcome domain in each study were sought (e.g. for all measures, time points, analyses), and if not, the methods used to decide which results to collect. | NA |
|  | 10b | List and define all other variables for which data were sought (e.g. participant and intervention characteristics, funding sources). Describe any assumptions made about any missing or unclear information. | NA |
| Study risk of bias assessment | 11 | Specify the methods used to assess risk of bias in the included studies, including details of the tool(s) used, how many reviewers assessed each study and whether they worked independently, and if applicable, details of automation tools used in the process. | 6 |
| Effect measures | 12 | Specify for each outcome the effect measure(s) (e.g. risk ratio, mean difference) used in the synthesis or presentation of results. | 6-7 |
| Synthesis methods | 13a | Describe the processes used to decide which studies were eligible for each synthesis (e.g. tabulating the study intervention characteristics and comparing against the planned groups for each synthesis (item #5)). | 6-7 |
|  | 13b | Describe any methods required to prepare the data for presentation or synthesis, such as handling of missing summary statistics, or data conversions. | 6-7 |
|  | 13c | Describe any methods used to tabulate or visually display results of individual studies and syntheses. | 6-7 |
|  | 13d | Describe any methods used to synthesize results and provide a rationale for the choice(s). If meta-analysis was performed, describe the model(s), method(s) to identify the presence and extent of statistical heterogeneity, and software package(s) used. | 6-7 |
|  | 13e | Describe any methods used to explore possible causes of heterogeneity among study results (e.g. subgroup analysis, meta-regression). | 6-7 |
|  | 13f | Describe any sensitivity analyses conducted to assess robustness of the synthesized results. | 6-7 |
| Reporting bias assessment | 14 | Describe any methods used to assess risk of bias due to missing results in a synthesis (arising from reporting biases). | 6-7 |
| Certainty assessment | 15 | Describe any methods used to assess certainty (or confidence) in the body of evidence for an outcome. | 6-7 |
| RESULTS | | |  |
| Study selection | 16a | Describe the results of the search and selection process, from the number of records identified in the search to the number of studies included in the review, ideally using a flow diagram. | 7-8, Figure 1 |
|  | 16b | Cite studies that might appear to meet the inclusion criteria, but which were excluded, and explain why they were excluded. | 7-8, Figure 1 |
| Study characteristics | 17 | Cite each included study and present its characteristics. | 7-9, appendix pp 22-25 |
| Risk of bias in studies | 18 | Present assessments of risk of bias for each included study. | 8, appendix pp 39-40 |
| Results of individual studies | 19 | For all outcomes, present, for each study: (a) summary statistics for each group (where appropriate) and (b) an effect estimate and its precision (e.g. confidence/credible interval), ideally using structured tables or plots. | appendix pp 27-32 |
| Results of syntheses | 20a | For each synthesis, briefly summarise the characteristics and risk of bias among contributing studies. | 10-13 |
|  | 20b | Present results of all statistical syntheses conducted. If meta-analysis was done, present for each the summary estimate and its precision (e.g. confidence/credible interval) and measures of statistical heterogeneity. If comparing groups, describe the direction of the effect. | 10-13, appendix pp 12-21, 33-36, Figures 2-4 |
|  | 20c | Present results of all investigations of possible causes of heterogeneity among study results. | 8-9 |
|  | 20d | Present results of all sensitivity analyses conducted to assess the robustness of the synthesized results. | appendix pp 14-21, 41-42 |
| Reporting biases | 21 | Present assessments of risk of bias due to missing results (arising from reporting biases) for each synthesis assessed. | Appendix pp 41-42 |
| Certainty of evidence | 22 | Present assessments of certainty (or confidence) in the body of evidence for each outcome assessed. | NA |
| DISCUSSION | | |  |
| Discussion | 23a | Provide a general interpretation of the results in the context of other evidence. | 13-16 |
|  | 23b | Discuss any limitations of the evidence included in the review. | 16-17 |
|  | 23c | Discuss any limitations of the review processes used. | 16-17 |
|  | 23d | Discuss implications of the results for practice, policy, and future research. | 17-18 |
| OTHER INFORMATION | | |  |
| Registration and protocol | 24a | Provide registration information for the review, including register name and registration number, or state that the review was not registered. | 4 |
|  | 24b | Indicate where the review protocol can be accessed, or state that a protocol was not prepared. | 4 |
|  | 24c | Describe and explain any amendments to information provided at registration or in the protocol. | 4 |
| Support | 25 | Describe sources of financial or non-financial support for the review, and the role of the funders or sponsors in the review. | 19 |
| Competing interests | 26 | Declare any competing interests of review authors. | 18 |
| Availability of data, code and other materials | 27 | Report which of the following are publicly available and where they can be found: template data collection forms; data extracted from included studies; data used for all analyses; analytic code; any other materials used in the review. | 18 |

## Text S2. Synthesis without meta-analysis in systematic reviews (SWiM) reporting guideline.

|  | **t** | **Synthesis Without Meta-analysis (SWiM) items: SWiM is intended to complement and be used as an extension to PRISMA** | | |
| --- | --- | --- | --- | --- |
|  | **SWiM reporting item** | | **Item description** | **Page in manuscript**  **where item is reported Other*** |
| **Methods** | | | | |
|  | 1 Grouping studies for synthesis | | 1a) Provide a description of, and rationale for, the groups used in the synthesis  (eg, groupings of populations, interventions, outcomes, study design)  1b) Detail and provide rationale for any changes made subsequent to the protocol in the groups used in the synthesis | 6-7 |
|  | 2 Describe the standardised metric and  transformation methods used | | Describe the standardised metric for each outcome. Explain why the metric(s) was chosen and describe any methods used to transform the intervention effects, as reported in the study, to the standardised metric, citing any methodological guidance consulted | 6-7 |
|  | 3 Describe the synthesis methods | | Describe and justify the methods used to synthesise the effects for each outcome when it was not possible to undertake a meta-analysis of effect estimates | 6-7 |
|  | 4 Criteria used to prioritise results for summary and synthesis | | Where applicable, provide the criteria used, with supporting justification, to select the particular studies, or a particular study, for the main synthesis or to draw conclusions from the synthesis (eg, based on study design, risk of bias assessments, directness in relation to the review question) | 6-7 |
|  | 5 Investigation of heterogeneity in reported effects | | State the method(s) used to examine heterogeneity in reported effects when it was not possible to undertake a meta-analysis of effect estimates and its extensions to investigate heterogeneity | 6-7 |
|  | 6 Certainty of evidence | | Describe the methods used to assess the certainty of the synthesis findings | 6-7 |
|  | 7 Data presentation methods | | Describe the graphical and tabular methods used to present the effects (eg, tables, forest plots, harvest plots)  Specify key study characteristics (eg, study design, risk of bias) used to order the studies, in the text and any tables or graphs, clearly referencing the studies included | 6-7 |
| **Results** | | | | |
|  | 8 Reporting results | | For each comparison and outcome, provide a description of the synthesised findings and the certainty of the findings. Describe the result in language that is consistent with the question the synthesis addresses, and indicate which studies contribute to the synthesis | 7-12, Figures 2-4 |
| **Discussion** | | | | |
|  | 9 Limitations of the synthesis | | Report the limitations of the synthesis methods used and/or the groupings used in the synthesis and how these affect the conclusions that can be drawn in relation to the original review question | 16-17 |
| PRISMA=Preferred Reporting Items for Systematic Reviews and Meta-Analyses.  *If the information is not provided in the systematic review, give details of where this information is available (eg, protocol, other published papers (provide citation details), or website (provide the URL)). | | | | |

## Text S3. Search Strategies

**Medline**

1. exp Respiratory Syncytial Virus Infections/ or exp Respiratory Syncytial Viruses/ or RSV.mp. or exp Respiratory Syncytial Virus, Human/

2. respiratory syncytial virus*.mp.

3. exp Pneumonia/ or exp Pneumonia, Viral/ or pneumonia.mp.

4. bronchiolitis.mp. or exp Bronchiolitis/ or exp Bronchiolitis, Viral/

5. exp Respiratory Tract Infections/ or respiratory infection*.mp.

6. respiratory disease*.mp. or exp Respiratory Tract Diseases/

7. exp Hospital Mortality/ or exp Mortality/ or mortality.mp.

8. exp Death/ or death*.mp. or exp "Cause of Death"/

9. exp Hospitalisation/ or hospitalisation*.mp.

10. burden.mp.

11. exp Epidemiology/ or epidemiology.mp.

12. exp Models, Statistical/ or statistical model*.mp.

13. model*.mp.

14. estimat*.mp.

15. modelling estimate*.mp.

16. 1 or 2

17. 3 or 4 or 5 or 6

18. 7 or 8 or 9 or 10 or 11

19. 12 or 13 or 14 or 15

20. 16 and 17 and 18 and 19

21. limit 20 to yr="1995 -2021"

limit 20 to (humans and yr="1995 - 2021")

**Global Health**

1 RSV.mp.

2 exp human respiratory syncytial virus/

3 respiratory syncytial virus*.mp.

4 exp community acquired pneumonia/ or pneumonia*.mp. or exp pneumonia/

5 bronchiolitis.mp. or exp bronchiolitis/

6 respiratory infection*.mp.

7 respiratory disease*.mp.

8 exp respiratory diseases/

9 mortality.mp. or exp mortality/

10 death*.mp. or exp death/ or exp "causes of death"/

11 (hospitalisation* or hospitalisation*).mp.

12 burden.mp.

13 exp epidemiology/ or epidemiology.mp.

14 model*.mp.

15 estimat*.mp.

16 1 or 2 or 3

17 4 or 5 or 6 or 7 or 8

18 9 or 10 or 11 or 12 or 13

19 14 or 15

20 16 and 17 and 18 and 19

21 limit 20 to (humans and yr="1995 - 2021")

**Embase**

1. exp Respiratory syncytial pneumovirus/ or RSV.mp. or exp respiratory syncytial virus infection

2. respiratory syncytial virus*.mp.

3. exp pneumonia/ or exp community acquired pneumonia/ or exp infectious pneumonia/ or pneumonia.mp. or exp virus pneumonia/

4. bronchiolitis.mp. or exp bronchiolitis/ or exp viral bronchiolitis/

5. exp respiratory tract infection/ or respiratory infection*.mp.

6. exp respiratory tract disease/ or respiratory disease*.mp.

7. exp hospital mortality/ or mortality.mp. or exp mortality/ or exp mortality rate/

8. exp death/ or death*.mp. or exp "cause of death"/

9. exp hospitalisation/ or hospitalisation*.mp.

10. burden.mp.

11. exp epidemiology/ or epidemiology.mp.

12. exp statistical model/ or statistical model*.mp. or exp model/

13. model*.mp.

14. estimat*.mp.

15. modelling estimate*.mp.

16. 1 or 2

17. 3 or 4 or 5 or 6

18. 7 or 8 or 9 or 10 or 11

19. 12 or 13 or 14 or 15

20. 16 and 17 and 18 and 19

21. limit 20 to (humans and yr="1995 - 2021")

## Text S4. Data extraction proforma

| **Domain** | **Extracted Information** |
| --- | --- |
| **Study characteristics** | First author  Year of publication  Country, Region  Time period  Age groups  Age bands reported  Inclusion and exclusion criteria  Case definition  Outcome measures  ICD-code |
| **Study model characteristics** | Modelling method  RSV activity proxy  Time lag  Climatic covariates  Pathogen covariates  Epidemic period definition |
| **Outcome measures** | For each hospitalisation or mortality outcome and age group  1. Rate estimate  2. 95% confidence intervals (if available) |

## Text S5. Description of multilevel model with random effects

In summary, we considered two levels in the multi-level meta-analysis model. Details were descripted below:

There are $i$ corresponding effect size estimates $T_{1}$, $T_{2}$…, $T_{i}$ with known sampling variances $\nu_{1}$, $\nu_{2}$ …, $\nu_{i}$. That is to say, the variance of an effect size estimate in the multi‐level model is:

${Var(T}_{i})= \nu_{i}+ \tau$	(1)

where τ is the level‐2 variance component. Separately, we assume that these effect size estimates $T_{i}$ are independently and normally distributed about $\vartheta_{i}$ with a mean of $\vartheta_{i}$ and variance $\nu_{i}$ .The model for the first level of the hierarchy (the within-study model) for effect size estimate $T_{i}$ is:

$T_{i}=\vartheta_{i}+\varepsilon_{i}$	(2)

where the error term $\varepsilon_{i}$ is normally distributed with a mean of zero and a variance vi.

In the second level of the hierarchy (the between-study model), the unknown effect‐size parameter $\vartheta_{i}$ varies around an overall mean, namely

$\vartheta_{i}=\beta_{0}+\eta_{i}$	(3)

where $\eta_{i}$ is a study-specific random effect that is normally distributed with a mean of zero and variance $\tau$ ($\tau$ > 0). In a single‐level notation, the model is written as:

$T_{i}=\beta_{0}+\eta_{i}+\varepsilon_{i}$	(4)

## Text S6. Modelling methods

**Regression model with RSV activity proxy**

Studies using generalized linear models (GLM) (including generalised additive models) incorporate a RSV activity proxy into the model and adopted various ways to control for seasonality or time trends. Other relevant covariates, such as time lag, other pathogens’ activity and climatic factors can also be included in the model. The basic formula of GLM is:

$$Y_{t}\sim D(\mu_{t})$$

$g\left( \mu_{t} \right)=\beta_{0}+\sum_{i=1}^{m} \beta_{i}{Virus}_{it}+f\left( t \right)+\sum_{j=1}^{k} q_{j}(z_{jt})$	[25]

where $Y_{t}$ represents the number of deaths/hospitalisations or the death/hospitalisation rate at time $t$ with a mean of $\mu_{t}$. D is the distribution of $\mu_{t}$. $g(.)$ represents the link function of $\mu_{t}$. β0 is the regression parameter associated with the baseline number of deaths/hospitalisations. ${Virus}_{it}$ is the $i$th viral activity proxy (i.e., RSV, flu etc.) at time $t$; $m$ is the number of proxies; $f$ is the function of the time variable $t$; $z_{jt}$ represents observed time-varying variables such as temperature and humidity at time $t$; k is the number of time-varying covariates; $q_{j}$ is a function of those variables.

**Rate-difference model**

The rate-difference method is simple and requires few assumptions. It has been used for many years to estimate influenza-associated disease burden. The average number of deaths or hospitalisations during periods assumed to have virus circulation (epidemic periods) and low or no virus circulation (baseline periods) are defined. Excess mortality or hospitalisation is calculated by subtracting numbers during baseline periods from observed deaths/hospital admissions during epidemic periods.

**Serfling-type model**

Studies using Serfling’s methods use cyclic regression to model the weekly proportion of attributable deaths or hospital admission and to define an epidemic threshold that is adjusted for seasonal effects. This method is well implemented in temperate countries where there are clear seasonal patterns of a given pathogen. However, this is not the case in tropical countries where seasonality is ill-defined for influenza and RSV.

## Text S7. Model characteristics

We identified 27 studies using regression models incorporating time-series data of a RSV activity proxy. Studies used various methods to control for unmeasured confounding. For example, all of studies used a polynomial function of time to control for trends in mortality or hospitalisation rate, or sine and cosine functions of time to control for the seasonality of mortality or hospitalisation rate. Among studies using regression models with an RSV activity proxy, ten studies [10,17,20-21,26-27,32,38,40,42] applied linear regression to estimate the RSV burden. Twelve studies [16,18,22-25,28,30-31,36-37,43] used a Poisson distribution and four studies [19,34,41,44] used a negative binomial distribution with an identity link or log link. Four different RSV activity proxies were identified. The most commonly used proxy (17/27) was the number of RSV-positive laboratory samples. 13 out of 27 studies included temperature or humidity as covariates. All regression models included influenza activity as a covariate and most studies (14/27) also incorporated other co-circulating pathogens. Eight studies also incorporated a time lag between predictor variables and outcome as an important covariate.

6 studies applied rate-difference models. All studies used different definitions of RSV-epidemic and non-epidemic periods (Table S3 in supplementary appendix). Broadly, half of studies (3/6) defined epidemic periods as starting when more than a given proportion of laboratory samples tested positive for 2 consecutive weeks and ending when less than this proportion was reported for 2 consecutive weeks. The remaining 3 studies defined RSV-epidemic periods as all weeks where a given proportion, or number within a cohort, of samples tested RSV-positive.

None of the studies used Serfling-type methods. 5 studies demonstrated multiple modelling approaches. Of these, only Emukule et al used two distinct model types (rate difference and negative binomial regression) [34]. The authors compared advantages and disadvantages of two methods and favoured a rate difference approach as their data were underpowered to estimate mortality using regression methods, despite its analytical advantage over the rate-difference method. Two studies produced estimates using rate difference methods with two different epidemic periods defined [35,47]. Both these authors emphasised the difficulties in defining epidemic periods. The estimations of virus-related burden strongly depended on the applied reference period, and estimates should, therefore, be viewed in rather large margins. Therefore, as these authors stated, it appears that the true RSV-associated excess mortality and hospitalisation probably lies within the estimations based on the peri-seasonal and summer baseline period. Two other studies compared RSV-activity proxies within Poisson regression models [18,31]. Both authors found using the number of laboratory samples positive for RSV as the more reliable RSV-activity proxy and age-specific counts shall be recommended as proxy variables if such data are available, as Chan et al indicated. Besides, only one study used Bayesian inference method and compared its performance with classical likelihood method [18]. The Bayesian inference method generally outperformed the classical likelihood estimation, particularly for RSV.

# Supplementary figures

## Figure S1. Geographical distribution of studies included.

## Figure S2. Median, IQR and ranges of reported estimates of rates of RSV-associated hospitalisation by country income level.

**Note:** ARI, acute respiratory infection; P&I, pneumonia and influenza. Y-axis is displayed in log scale. Number at the bottom of each panel indicates number of data-points, if studies reported estimates for multiple years or seasons, then this study contributes to multiple data points.

## Figure S3. Annual rates of RSV-associated ARI hospitalisation per 100,000 over time by study and age group.

**Note:** Y-axis is displayed in log scale. Children group includes three age groups (i.e., <1 year, 1-4 years and 5-17 years). Adult group includes four age groups (i.e., 18-49 years, 50-64 years, 65-74 years and 75+ years).

## Figure S4. Annual rates of RSV-associated mortality per 100,000 over time by study and age group.

**Note:** Y-axis is displayed in log scale. Children group includes three age groups (i.e., <1 year, 1-4 years and 5-17 years). Adult group includes four age groups (i.e., 18-49 years, 50-64 years, 65-74 years and 75+ years).

## Figure S5. Forest plots of meta-analysis of age-specific RSV-associated hospitalisation rates in high income countries by age group and case definition.

A.RSV-C&R hospitalisation rate for 65-74 years

B.RSV-C&R hospitalisation rate for 75+ years

C.RSV-C&R hospitalisation rate for 65+ years

D.RSV-CRD hospitalisation rate for 75+ years

E.RSV-ARI hospitalisation rate for <1 year

F.RSV-ARI hospitalisation rate for 1-4 years

G.RSV-ARI hospitalisation rate for 75+ years

H.RSV-ARI hospitalisation rate for all ages

I.RSV-ALRI hospitalisation rate for <1 year

J.RSV-ALRI hospitalisation rate for 1-4 years

K.RSV-P&I hospitalisation rate for <1 year

L.RSV-P&I hospitalisation rate for 1-4 years

**Note:** C&R, circulatory and respiratory; ARI, acute respiratory infection; ALRI, acute lower respiratory infection; CRD, chronic respiratory infection; P&I, pneumonia and influenza.

## Figure S6. Forest plots of meta-analysis of age-specific RSV-associated mortality rates in upper-middle income countries by age group and case definition.

A.RSV associated all-cause mortality for 1-4 years

B.RSV associated all-cause mortality for 5-17 years

C.RSV associated all-cause mortality for 18-49 years

D.RSV associated all-cause mortality for 65+ years

E.RSV associated ARI mortality for <1 year

F.RSV associated ARI mortality for 1-4 years

G.RSV associated ARI mortality for 5-17 years

H.RSV associated ARI mortality for 18-49 years

I.RSV associated ARI mortality for <5 years

J.RSV associated ARI mortality for 5+ years

K.RSV associated ARI mortality for all ages

L.RSV associated P&I mortality for <1 year

M.RSV associated P&I mortality for 1-4 years

N.RSV associated P&I mortality for 5-17 years

O.RSV associated P&I mortality for 18-49 years

P.RSV associated P&I mortality for <5 years

Q.RSV associated P&I mortality for 5+ years

## Figure S7. Comparison of median estimates (main analysis) and meta estimates (“sensitivity analysis”) for RSV-associated hospitalisation and mortality rates.

**Note:** X and Y-axis are displayed in log scale. Each point represents pooled estimates in certain case definition, age group and country income level.

# Supplementary tables of results

## Table S1. Risk of bias and quality assessment for included studies

| **Study** | **Was the activity of other viruses other than influenza considered in model? (no/unclear – 0 point; yes – 1 point)** | **Was time lag between RSV activity and outcome considered in models? (no/unclear – 0 point; yes – 1 point)** | **Was age-stratified or all-ages virological data used to guide the timing of epidemics in models? (no/unclear – 0 point; yes – 1 point)** | **Was the hospitalisation or mortality ICD-code consistent during the study period? (no/unclear – 0 point; yes – 1 point)** | **Did the study span five years or more? (no – 0 point; yes – 1 point)** | **Was weekly virological surveillance data used? (no – 0 point; yes – 1 point)** | **Total points** |
| --- | --- | --- | --- | --- | --- | --- | --- |
| Yang, 2011 [16] | yes | no | yes | yes | no | yes | 4 |
| Matias, 2017 [40] | no | no | yes | yes | yes | yes | 4 |
| Mullooly, 2007 [45] | no | yes | yes | yes | no | yes | 4 |
| Zhou, 2012 [41] | no | no | yes | yes | yes | yes | 4 |
| Chan, 2014 [18] | yes | yes | yes | yes | yes | yes | 6 |
| Cromer, 2017* [19] | no | no | yes | yes | yes | yes | 4 |
| Fleming, 2015* [10] | no | yes | no | no | yes | yes | 3 |
| Müller-Pebody, 2006 [42] | yes | no | yes | yes | no | yes | 4 |
| Reeves, 2016 [20] | yes | no | yes | yes | yes | yes | 5 |
| Jansen, 2007* [35] | no | yes | yes | yes | yes | yes | 5 |
| Kyeyagalire, 2014 [22] | yes | no | no | yes | yes | no | 3 |
| Schanzer, 2006 [23] | yes | no | yes | yes | yes | yes | 5 |
| Schanzer, 2008 [24] | yes | no | yes | yes | yes | yes | 5 |
| Schanzer, 2018 [25] | yes | no | yes | yes | yes | yes | 5 |
| Učakar, 2013 [47] | yes | yes | yes | yes | yes | yes | 6 |
| Elliott Bosco,2021 [44] | no | yes | yes | no | yes | yes | 4 |
| Sharp,2021 [26] | yes | yes | yes | yes | yes | yes | 6 |
| Goldstein,2015 [27] | no | yes | no | yes | yes | yes | 4 |
| Cromer,2013* [38] | yes | yes | yes | yes | yes | yes | 4 |
| Matias, 2014 [17] | no | no | yes | no | yes | yes | 3 |
| Thompson, 2003 [28] | no | no | yes | no | yes | yes | 3 |
| Fleming, 2005 [29] | no | yes | yes | yes | yes | yes | 5 |
| Hardelid, 2013 [30] | no | yes | yes | unclear | yes | yes | 4 |
| Markov, 2007 [31] | no | no | yes | yes | yes | yes | 4 |
| Taylor, 2016 [21] | no | no | yes | no | yes | yes | 3 |
| Nicholson, 1996 [32] | yes | no | no | unclear | yes | yes | 3 |
| Cohen, 2018 [33] | no | no | yes | yes | no | yes | 3 |
| Emukule, 2017 [34] | no | no | yes | yes | yes | no | 3 |
| Karstaedt, 2009 [46] | no | no | no | unclear | no | yes | 1 |
| Tempia, 2014 [43] | no | no | yes | yes | yes | no | 3 |
| Tempia, 2015 [36] | no | no | yes | yes | yes | no | 3 |
| van Asten, 2012 [37] | yes | yes | yes | unclear | yes | yes | 5 |

## Table S2. Summary of modelling studies reporting RSV-associated hospitalisation.

| **Author, Year** | **Location** | **Time period** | **Age bands reporting** | **Modelling method** | **Case definition used** | **ICD code(s)** |
| --- | --- | --- | --- | --- | --- | --- |
| Yang, 2011 [16] | China, Hong Kong | Oct 2003 – Sep 2006 | <1y, 1-<2y, 2-4y, 5-9y, 10-17y | Poisson regression | Acute respiratory disease | ICD9 |
| Matias, 2017 [40] | US | Oct 1997 – Mar 2009 | 0-4y, 5-17y, 18-49y, 50-64y, 65-74y, >74y | Linear regression | P&I, respiratory broad, respiratory, cardiorespiratory disease or sepsis | ICD9, ICD10 |
| Mullooly, 2007 [45] | US | 1996 - 2000 | 18-49y, 50-64y, ＞64y | Rate difference | P&I, C&R, acute cerebrovascular disease (CVD), cardiac disease | ICD9 |
| Zhou, 2012[41] | US | 1993 - 2008 | 0-11m, 1-4y, 5-49y, 50-64y, >64y | Negative binomial regression | Cardiorespiratory | ICD9 |
| Chan, 2014[18] | China, Hong Kong | Oct 2003 – Sep 2010 | <1y, 1-5y, 6-17y | Poisson regression; Bayesian inference method | Acute respiratory disease | ICD9 |
| Cromer, 2017* [19] | UK | 2000 - 2008 | <6m, 6m-4y, <5y | Negative binomial regression | Acute respiratory illness | ICD10 |
| Fleming, 2015* [10] | UK | 1997 -2009 | 18-49y, 50-64y, 65-74y, >74y | Linear regression | Respiratory disease, COPD, cardiorespiratory disease, bronchitis/bronchiolitis | ICD9, ICD10 |
| Müller-Pebody, 2006 [42] | UK | Apr 1995 – Apr 1998 | >64y | Linear regression | Lower respiratory infection | ICD10 |
| Reeves, 2016 [20] | UK | Jul 2007 – Jul 2012 | <6m, 6-11m, 1-4y | Linear regression | Respiratory tract infection: bronchiolitis, pneumonia, bronchitis, LRTI and URTI | ICD10 |
| Jansen, 2007* [35] | Netherlands | 1997 - 2003 | 0-1y, 2-4y, 5-17y, 18-49y, 50-64y, >64y | Rate difference | Acute upper respiratory disease, acute/chronic lower respiratory disease, cardiovascular disease, cerebrovascular disease, bacterial disease or other condition | ICD9 |
| Kyeyagalire, 2014 [22] | South Africa | 2007 - 2012 | <1y, 1-4y, 5-19y, 20-44y, 45-64y, 65-74y, >74y | Poisson regression | All-respiratory, P&I | ICD10 |
| Schanzer, 2006 [23] | Canada | Sep 1994 – Aug 2000 | 0-19y | Poisson regression | Multiple/unspecified ARI, bronchitis/bronchiolitis, pneumonia, asthma, other primary respiratory, all-respiratory | ICD9 |
| Schanzer, 2008 [24] | Canada | Sep 1994– Aug 2000 | 20-49y, 50-64y, >64y | Poisson regression | All-cause, ARI, pneumonia, Influenza, asthma, other primary respiratory, secondary respiratory diagnosis, all-respiratory | ICD9 |
| Schanzer, 2018 [25] | Canada | Sep 2004 – Aug 2014 | <2y, 2-4y, 5-16y, 17-44y, 45-64y, >64y | Poisson regression | All-respiratory primary diagnosis | ICD10 |
| Učakar, 2013 [47] | Slovenia | 2006 - 2011 | <6y | Rate difference | Acute lower respiratory tract infection: pneumonia, acute bronchitis or bronchiolitis | ICD10 |
| Elliott Bosco,2021 [44] | US | 2011-2017 | 65-74y, 75-84y, >84y | Negative binomial regression | Cardiorespiratory Hospitalisation | ICD9, ICD10 |
| Sharp,2021 [26] | UK | Aug 2010 – Jul 2017 | 65-74y, >74y | linear multiple regression | Cardiorespiratory disease, Respiratory disease, Influenza and Pneumonia, Bronchitis, Chronic respiratory disease | ICD10 |
| Goldstein,2015 [27] | US | 2003-2011 | <1y, 1–4y, 5–17y, 18–49y, 50–64y, 65–74y, >74y | Linear regression | All respiratory, P&I, CLRD, circulatory hospitalisations | ICD9 |
| Cromer,2013* [38] | UK | 2000-2007 | <6m, 6m-4y, 5-14y, 15-44y, 45-64y, >64y | Generalized linear regression | ARI hospitalisation | ICD10 |

**Note:** US=United States of America; UK=United Kingdom; Y=year; P&I, pneumonia and influenza; C&R, circulatory and respiratory; ARI, acute respiratory infection; COPD, chronic obstructive pulmonary disease; CLRD, chronic lower respiratory disease; URTI, upper respiratory tract infection; ICD= the International Classification of Diseases. *Studies reporting both hospitalisation and mortality data.

## Table S3. Summary of modelling studies reporting RSV-associated mortality.

| **Author, Year** | **Location** | **Time period** | **Age bands reporting** | **Modelling method** | **Case definition used** | **ICD code(s)** |
| --- | --- | --- | --- | --- | --- | --- |
| Matias, 2014 [17] | US | Oct 1997 – Mar 2009 | all ages | Linear regression | P&I death, Cardiorespiratory, Respiratory broad | ICD9, ICD10 |
| Thompson, 2003 [28] | US | Jul 1990 – Jun 1999 | 0-11m, 1-4y, 5-49y, 50-64y, >64y | Poisson regression | P&I, C&R and all-cause death | ICD9, ICD10 |
| Cromer, 2017* [19] | UK | 2000 - 2008 | <6m, 6m-4y, <5y | Negative binomial regression | ARI death | ICD10 |
| Fleming, 2015* [10] | UK | 1997 - 2009 | 18-49y, 50-64y, 65-74y, >74y | Linear regression | Respiratory disease, COPD, C&R and bronchitis/bronchiolitis death | ICD9, ICD10 |
| Fleming, 2005 [29] | UK | Aug 1989 – May 2000 | 1-12m, 1-4y, 5-9y, 10-14y, >15y, all ages | Rate difference | All-cause and respiratory death | ICD9 |
| Hardelid, 2013 [30] | UK | Jan 1999 – May 2010 | <15y, 15-44y, 45-74y, >74y | Multivariable regression (Poisson regression <45 years and negative binomial regression >45 years) | All-cause death | ___ |
| Markov, 2007 [31] | UK | Jan 1993 – Dec 2000 | <16y | Poisson regression | Pneumonia death | ICD9 |
| Taylor, 2016 [21] | UK | 1996 - 2009 | <6m, 6-23m, 2-4y, 5-17y | Linear regression | Respiratory disease, P&I and bronchitis/bronchiolitis death | ICD9, ICD10 |
| Nicholson, 1996 [32] | UK | 1975 - 1990 | all ages | Linear regression | URTI-associated death | ___ |
| Cohen, 2018 [33] | South Africa | 2009 - 2013 | <1y, 1-4y, 5-19y, 20-44y, 45-64y, 65-74y, >74y, <5y, >4y | Generalised additive regression | All-cause, all-circulatory, all-respiratory and P&I death | ICD10 |
| Emukule, 2017 [34] | Kenya | Jan 2007 – Dec 2013 | <5y, 5-49y, >49y | Negative binomial regression; rate difference | All-cause, respiratory, pulmonary TB and HIV/AIDS death | ICD10 |
| Jansen, 2007* [35] | Netherlands | 1997 - 2003 | 0-1y, 2-4y, 5-17y, 18-49y, 50-64y, >64y | Rate difference | Acute/chronic lower respiratory, cardiovascular, cerebrovascular and bacterial disease death | ICD9 |
| Karstaedt, 2009 [46] | South Africa | Jan 1997 – Dec 1999 | <65y, >64y | Rate difference | All-cause death | ___ |
| Tempia, 2014 [43] | South Africa | 1998 - 2009 | <1y, 1-4y | Poisson regression | All-respiratory and P&I death | ICD10 |
| Tempia, 2015 [36] | South Africa | 1998 - 2009 | 5-19y, 20-44y, 45-64y, 65-74y, >74y | Poisson regression | All-cause, all-respiratory, all-circulatory and P&I death | ICD10 |
| van Asten, 2012 [37] | Netherlands | Jan 1999 – Dec 2007 | 65-74y, 75-84y, >84y | Poisson regression | All-cause death | ___ |
| Cromer,2013* [38] | UK | 2000-2007 | <6m, 6m-4y, 5-14y, 15-44y, 45-64y, >64y | Generalized linear regression | All-cause mortality | ICD10 |

**Note:** US=United States of America; UK=United Kingdom; Y=year; P&I, pneumonia and influenza; C&R, circulatory and respiratory; ARI, acute respiratory infection; COPD, chronic obstructive pulmonary disease; URTI, upper respiratory tract infection. ICD= the International Classification of Diseases. * Studies reporting both hospitalisation and mortality data.

## Table S4. Definitions of RSV-epidemic periods used in studies applying rate-difference methods.

| **Study (author, year)** | **Epidemic period definition** |
| --- | --- |
| Mullooly, 2007 [45] | Weeks ≥10% of samples tested are positive for RSV |
| Fleming, 2005 [10] | Weeks ≥200 RSV positive samples in a cohort of children aged 0-1 year |
| Emukule, 2017 [34] | Weeks ≥12% samples tested are positive for RSV |
| Jansen, 2007 [35] | Starts when 2 consecutive weeks contain ≥5% season total of RSV cases |
| Karstaedt, 2009 [46] | Starts when 2 consecutive weeks contain ≥3% of annual total of tested samples RSV positive |
| Ucakar, 2013 [47] | Starts when for 2 consecutive weeks ≥10% tested samples are positive for RSV |

## Table S5. Summary of the reported average annual estimates of RSV all-cause mortality rate (per 100,000).

| **Age group (years)** | **Cohen**  **2018 (GAR)** | **Tempia**  **2015 (PR)** | **Fleming 2005**  **(RD)** | **Tempia**  **2014**  **(PR)** | **Fleming 2015**  **(LR)** | **Jansen 2007 (RD)** | **Thompson 2003**  **(****PR)** | **van Asten 2012**  **(PR)** | **Emukule 2017**  **(RD)** | **Emukule 2017 (NBR)** | **Markov2007**  **(PR)** | **Taylor 2016**  **(LR)** | **Matias2014**  **(LR)** | **Karstaedt 2009**  **(RD)** | **Schanzer 2018**  **(PR)** |
| --- | --- | --- | --- | --- | --- | --- | --- | --- | --- | --- | --- | --- | --- | --- | --- |
| **<1** | 143.3 (0, 195.4) |  | 8.4 |  |  |  | 5.4 |  |  |  | 15.1(9.4,20.9) | 6 |  |  |  |
| **1-4** | 7 (3.9, 17) |  | 0.9 |  |  |  | 0.9 |  |  |  |  | 0 |  |  |  |
| **<5** | 23.4 (14.2, 31.6) |  |  | 10.3 |  |  |  |  | 90.3 (63.8, 127.8) | 32.6 (0, 397.1) |  |  | 0.8 |  |  |
| **5-17** | 3.7 (2.5, 8.4) | 0.4 (0.2, 0.6) | 0.2 |  |  |  |  |  |  |  |  |  | 0.1 |  |  |
| **18-49** | 9.9 (5.5, 26) | 2.4 (0.4, 4.7) | 0 |  | 0 | 0.1 (-0.4, 0.5) |  |  |  |  |  |  | 0.3 |  |  |
| **50-64** | 15.7 (10.4, 53.6) |  | 6 |  | 6 | 1.9 (-0.1, 3.7) | 7.8 |  |  |  |  |  | 2.2 |  |  |
| **65+** |  |  | 88 |  | 88 | 52.1 (46.1, 58.2) | 29.6 | 622 |  |  |  |  |  | 91.2 |  |
| **65-74** | 22 (14.5,67.6) |  | 29 |  | 29 |  |  |  |  |  |  |  | 8.7 |  |  |
| **75+** | 81.7 (72.7,339.5) |  | 155 |  | 155 |  |  |  |  |  |  |  | 46.4 |  |  |
| **all ages** | 13.2(6.4,33.8) |  | 15 |  | 15 |  | 6.6 |  | 19.7 (14.7, 26.5) | 17.1 (0, 111.5) |  |  | 3.9 | 7.4 | 0.6 (-0.1,1.3) |

**Note:** GAR-Generalised additive regression; PR- Poisson regression; RD-Rate difference; LR-Linear regression; NBR- negative binomial regression;

## Table S6. Summary of the reported average annual estimates of RSV-C&R hospitalisation and mortality rate (per 100,000).

|  | **RSV-C&R hospitalisation** | | | | | | **RSV-C&R mortality** | | |
| --- | --- | --- | --- | --- | --- | --- | --- | --- | --- |
| **Age group (years)** | **Matias, 2017 (LR)** | **Mullooly, 2007 (RD)** | **Zhou, 2012**  **(NBR)** | **Fleming, 2015 (LR)** | **Elliott Bosco, 2022 (NBR)** | **Sharp,2021 (LR)** | **Matias, 2014 (LR)** | **Thompson, 2003 (PR)** | **Fleming, 2015 (LR)** |
| **<1** |  |  | 2345 (2219,2525) |  |  |  |  | 5.3 |  |
| **1-4** |  |  | 178.2 (155,230) |  |  |  |  | 0.2 |  |
| **<5** |  |  |  |  |  |  | 0.7 |  |  |
| **5-17** |  |  |  |  |  |  | 0.1 |  |  |
| **18-49** |  | 2 (-7,12) |  | 1 |  |  | 0.7 |  | 1 |
| **50-64** |  | 9 (-10,28) | 12.8 (2.4,73.9) | 18 |  |  | 4 | 4.7 | 8 |
| **65+** |  | 106 (42,170) | 86.1 (37.3,326) | 112 | 122 (93,151) |  |  | 26.5 | 113 |
| **65-74** |  |  |  | 56 | 64 (24,103) | 61 (42,80) | 13.2 |  | 37 |
| **75+** |  |  |  | 174 |  | 232 (162,301) | 68.1 |  | 198 |
| **all ages** | 49 |  | 55.3 (44.4,107) | 69 |  |  | 6 | 43 | 20 |

**Note:** GAR-Generalised additive regression; PR- Poisson regression; RD-Rate difference; LR-Linear regression; NBR- negative binomial regression.

## Table S7. Summary of the reported average annual estimates of RSV-ARI hospitalization rate (per 100,000).

| **Age group (years)** | **Chan**  **2014**  **(PR)** | **Reeves**  **2016**  **(LR)** | **Kyeyagalire**  **2014**  **(PR)** | **Cromer**  **2017**  **(NBR)** | **Matias**  **2017**  **(LR)** | **Fleming**  **2015**  **(LR)** | **Schanzer**  **2008**  **(PR)** | **Mullooly**  **2007**  **(RD)** | **Yang 2011 (PR)** | **Taylor 2016**  **(LR)** | **Goldstein 2015**  **(LR)** | **Schanzer 2018**  **(PR)** | **Sharp 2021**  **(LR)** |
| --- | --- | --- | --- | --- | --- | --- | --- | --- | --- | --- | --- | --- | --- |
| **<1** | 2501.2 (2012.4, 2964) | 3510 (3290, 3890) | 7601 (4312, 10817) | 4400 (4380,4410) |  |  |  |  | 1757 | 4184 |  |  |  |
| **1-4** | 468 (265.2, 655.2) | 531 (446, 659) | 1182 (704, 1643) | 900 (890,900) | 514 |  |  |  | 179 | 114 | 116.7 (69.6,167) | 106.2 (79,134) |  |
| **5-17** | 15.6 (0, 36.4) |  | 57 (26, 88) |  | 0.5 |  |  |  | 43 | 0.5 |  | 5.3 (-2.4,12.9) |  |
| **18-49** |  |  |  |  | 9 | 4 | 10 |  |  |  |  | 1.2 (-1.3,3.6) |  |
| **50-64** |  |  |  |  | 28 | 40  30 | 23 |  |  |  |  | 5 (-0.2,10.1) |  |
| **65+** |  |  |  |  | 258 | 156 | 108 | 51 (7, 95) |  |  |  | 52.7 (25,80) |  |
| **65-74** |  |  |  |  | 84 | 86 |  |  |  |  |  |  | 71 (52,90) |
| **75+** |  |  |  |  | 258 | 234 |  |  |  |  | 174.9 (43.6,316) |  | 251 (186,316) |
| **all ages** |  |  | 223 (128, 317) |  | 64 | 83 | 27.2 (21.5,32.9) |  |  |  |  |  |  |

**Note:** GAR-Generalised additive regression; PR- Poisson regression; RD-Rate difference; LR-Linear regression; NBR- negative binomial regression.

## Table S8. Summary of the reported average annual estimates of RSV-ARI mortality rate (per 100,000)

| **Age group (years)** | **Cohen 2018 (GAR)** | **Tempia 2014**  **(PR)** | **Tempia 2015 (PR)** | **Fleming 2005**  **(RD)** | **Fleming 2015 (LR)** | **Taylor 2016**  **(LR)** | **Matias 2014**  **(LR)** | **Emukule 2017**  **(RD)** | **Emukule 2017**  **(NBR)** | **Cromer**  **2017**  **(NBR)** |
| --- | --- | --- | --- | --- | --- | --- | --- | --- | --- | --- |
| **<1** | 42 (0, 63.9) | 35 (17, 54) |  | 2.9 |  |  |  |  |  | 2.48 (2.38,2.58) |
| **1-4** | 5 (2.5, 7.9) | 4 (1, 7) |  | 0.3 |  | 3 |  |  |  | 0.66 (0.64,0.68) |
| **<5** | 12.7 (2,19.4) | 10 (5, 15) |  |  |  |  | 0.8 | 38.5 (0,109.9) | 38.5 (0, 109.9) | 0.85 (0.83,0.87) |
| **5-17** | 0.6 (0.3, 1.4) | 0.3 (0.1, 0.4) |  | 0.1 |  | 0 | 0.1 |  |  |  |
| **18-49** | 1.2 (0.4, 4.6) | 2.1 (1.7, 2.5) | 2.1 | 1 | 1 |  | 0.3 |  |  |  |
| **50-64** | 4.6 (2.7, 14.3) |  |  |  | 6 |  |  | 5.9 (0,29) |  |  |
| **65+** |  |  |  |  | 88 |  |  |  |  |  |
| **65-74** | 4.2 (0,34.3) |  |  |  | 29 |  |  |  |  |  |
| **75+** | 16 (13, 88.1) |  |  |  | 155 |  |  |  |  |  |
| **all ages** | 7.8 (3.9, 10.9) |  |  |  |  |  |  |  | 7.3 (0, 27.3) |  |

**Note:** GAR-Generalised additive regression; PR- Poisson regression; RD-Rate difference; LR-Linear regression; NBR- negative binomial regression.

## Table S9. Summary of the reported average annual estimates of RSV-ALRI hospitalisation and mortality rate (per 100,000)

|  | **RSV-ALRI hospitalisation** | | | | | | | **RSV-ALRI mortality** | | |
| --- | --- | --- | --- | --- | --- | --- | --- | --- | --- | --- |
| **Age group (years)** | **Fleming, 2015(LR)** | **Muller-Pebody, 2006(LR)** | **Reeves, 2016(LR)** | **Taylor, 2016(LR)** | **Jansen, 2007(RD)** | **Ucakar, 2013(RD)** | **Sharp,2021(LR)** | **Fleming, 2015(LR)** | **Taylor, 2016(LR)** | **Jansen, 2007(RD)** |
| **<1** |  |  | 3020 (2900,3230) | 3760 | 608.2 (597,620) |  |  |  |  |  |
| **1-4** |  |  | 90(75,84) | 59 | 34.6 (31,38.2) |  |  |  |  |  |
| **5-17** |  |  |  | 3 | 1.8 (1.1,2.5) |  |  |  | 0 |  |
| **18-49** | 2 |  |  |  |  |  |  | 0 |  |  |
| **50-64** | 7 |  |  |  |  |  |  | 0 |  |  |
| **65+** | 46 | 128 |  |  |  |  |  | 3 |  | 37 (33.5,40.4) |
| **65-74** | 19 |  |  |  |  |  | 12 (8,16) | 1 |  |  |
| **75+** | 75 |  |  |  |  |  | 58 (42,74) | 5 |  |  |
| **all ages** | 47 |  |  |  |  |  |  | 1 |  |  |

**Note:** GAR-Generalised additive regression; PR- Poisson regression; RD-Rate difference; LR-Linear regression; NBR- negative binomial regression.

## Table S10 Summary of the reported average annual estimates of RSV-P&I hospitalisation and mortality rate (per 100,000).

|  | **RSV-P&I hospitalisation** | | | | | | | | | **RSV-P&I mortality** | | | | | | |
| --- | --- | --- | --- | --- | --- | --- | --- | --- | --- | --- | --- | --- | --- | --- | --- | --- |
| **Age group (years)** | **Matias, 2017 (LR)** | **Mullooly, 2007 (RD)** | **Fleming, 2015 (LR)** | **Reeves, 2016 (LR)** | **Taylor, 2016(LR)** | **Kyeyagalire, 2014**  **(PR)** | **Ucakar, 2013 (RD)** | **Sharp,2021 (LR)** | **Goldstein, 2015 (LR)** | **Matias, 2014 (LR)** | **Thompson, 2003 (PR)** | **Fleming, 2015 (LR)** | **Taylor, 2016 (LR)** | **Cohen, 2018 (GAR)** | **Tempia, 2014 (PR)** | **Tempia, 2015 (PR)** |
| **<1** |  |  |  | 104 (87,126) | 60 | 3055 (1732,4345) |  |  | 183.8 (115,254) |  | 3.1 |  | 2 | 30.4 (0,42.1) | 33 (18,52) |  |
| **1-4** |  |  |  | 99 (88,109) | 48 | 457 (272,635) |  |  | 71.9 (38.5,106) |  | 0.1 |  | 0 | 3.3 (2.2,5.8) | 3 (1,5) |  |
| **<5** |  |  |  |  |  |  | 1071 |  |  | 0.5 |  |  |  | 8.9 (1.8,13.3) | 9 (4,14) |  |
| **5-17** |  |  |  |  | 4 | 21 (9,32) |  |  | -1.3 (-9.9,7.4) | 0 |  |  | 0 | 0.6 (0.1,1) |  | 0.2 (0.1,0.3) |
| **18-49** |  | 2 (-6,10) | 2 |  |  |  |  |  | 6.2 (1.1,11.3) | 0.2 |  | 0 |  | 0.9 (0.2,4.1) |  | 1.4 (0.7,2.2) |
| **50-64** |  | 10 (-10,30) | 7 |  |  |  |  |  | 6.1 (-9.6,22.8) | 0.8 | 0.5 | 4 |  | 2.4 (1.1,6.8) |  |  |
| **65+** |  | 51 (7,95) | 49 |  |  |  |  |  |  |  | 7.2 | 66 |  |  |  |  |
| **65-74** |  |  | 20 |  |  |  |  | 25 (17,33) | 4.4 (-32,38) | 4.3 |  | 17 |  | 5.1 (1.1,17.7) |  |  |
| **75+** |  |  | 80 |  |  |  |  | 118 (84,151) | 56.8 (-25,143) | 27.4 |  | 121 |  | 15.7 (6.4,67.4) |  |  |
| **all ages** | 20 |  | 15 |  |  | 86 (50,125) |  |  |  | 2.2 | 1 | 11 |  | 2.2 (0.6,5.9) |  |  |

**Note:** GAR-Generalised additive regression; PR- Poisson regression; RD-Rate difference; LR-Linear regression; NBR- negative binomial regression

## Table S11. Methods for estimating RSV-associated hospitalisation and mortality.

| **Methods** | **Description** | **Limitation and biases** |
| --- | --- | --- |
| 1. Prospective cohort | Comprehensive systematic collection of epidemiological information and biological samples from participants to provide a direct estimate of RSV-attributable hospitalisation/mortality incidence | Resource intensive to establish and maintain and therefore tend to cover limited periods |
| 2. Data-linkage | Linkage of routine hospital data/death certificates to laboratory records to derive estimate of RSV-attributable hospitalisation or death | Relies on robust administrative data and viral surveillance. Therefore, not applicable in most countries |
| 3. Multiplicative method | Respiratory mortality multiplier defined based on case-fatality rate data from a range of settings. Applied to larger populations to derive estimates of RSV-attributable hospitalisation or death | Limited data available from low-income settings in current approaches |
| 4. Statistical modelling techniques | Estimates RSV-attributable hospitalisation/mortality through comparing selected-cause hospitalisation/mortality data during epidemic periods and non-epidemic periods; applies a variety of models which may or may not be strengthened by viral surveillance data | May be confounded by rises in hospitalisation/mortality due to other causes |

## Table S12. Modelling approaches for estimation of RSV-associated hospitalisation and mortality.

| **Approaches** | **Data requirements** | **Statistical method** | **Strengths** | **Weaknesses** |
| --- | --- | --- | --- | --- |
| 1. Rate difference | i) Weekly/monthly morbidity/mortality data.  ii) Viral surveillance data | Arbitrary defining of epidemic periods to attribute excess cases to RSV | Can be used in regions with varied RSV-seasonality and less than 5 seasons baseline data | Estimates differ with epidemic thresholds.  Can’t account for seasonal factors (e.g., temperature and humidity) |
| 2. Regression model with a RSV-activity proxy | i) Weekly/monthly morbidity/mortality data.  ii) Viral surveillance data | Regression used to model the time-series patterns of RSV health outcomes due to seasonality, and over time, adjusting for confounders | Accounts for changes in population size over time and able to incorporate other variables (e.g. co-circulating pathogens, temperature, humidity) | Requires robust viral surveillance data and at least 5 years of data for stable estimates |
| 3. Serfling-type methods | i) Well-defined RSV seasonality  ii) Weekly/monthly morbidity/mortality data. | Uses expected RSV seasonality during baseline period to identify a proportion of health outcomes which are RSV-attributable | Robust viral surveillance data not required. | Not applicable for countries lacking well defined RSV -seasonality (e.g. tropical/sub-tropical) |

## Table S13. Median and range of reported estimates of ALRI, P&I, C&R and ARI hospitalisation associated with RSV per 100,000 by age group in high income countries.

|  | **RSV-ALRI hospitalisation** | | | | **RSV-P&I hospitalisation** | | | | | **RSV-C&R hospitalisation** | | | | | **RSV-ARI hospitalisation** | | | | |
| --- | --- | --- | --- | --- | --- | --- | --- | --- | --- | --- | --- | --- | --- | --- | --- | --- | --- | --- | --- |
| **Age group (years)** | **median** | **Range** | **[P25, P75]** | **No.** | **median** | **range** | **[P25, P75]** | **No.** | **median** | | **range** | **[P25, P75]** | **No.** | **median** | | **range** | **[P25, P75]** | **No.** |  |
| **<1** | 3560.0 | [608.2, 13317.2] | [3012.5, 6626.1] | 12 | 157.5 | [54, 3055] | [68.5, 199.5] | 15 | 2227.7 | | [1817.2, 3150.7] | [2029.5, 2640.4] | 15 | 2235.7 | | [1353, 7601] | [1779.1, 3552.5] | 20 |  |
| **1-4** | 90.5 | [34.6, 2366] | [70, 852.8] | 12 | 78.5 | [48, 457] | [67.9, 95.0] | 15 | 176.6 | | [139.5, 260.4] | [158.5, 188.0] | 15 | 171.0 | | [96.4, 4470] | [115.3, 515.5] | 23 |  |
| **5-17** | 2.4 | [1.8, 3] | [2.1, 2.7] | 2 | 12.5 | [4, 21] | [8.3, 16.8] | 2 | __ | | __ | __ | __ | 1.6 | | [0.5, 57] | [1.3.18.5] | 16 |  |
| **18-49** | 2.0 | [2, 2] | [2, 2] | 1 | 5.9 | [2, 7.2] | [5.2, 6.5] | 10 | __ | | __ | __ | __ | 11.0 | | [1.2, 14.2] | [9.8, 12.7] | 12 |  |
| **50-64** | 7.0 | [7, 7] | [7, 7] | 1 | 6.5 | [5.1, 10] | [5.7, 7.0] | 10 | 12.6 | | [7.3, 19.9] | [9.6, 15.4] | 17 | 27.2 | | [5, 31.9] | [23.9, 29.5] | 12 |  |
| **65-74** | 15.5 | [12, 19] | [13.8, 17.3] | 2 | 4.7 | [3.7, 25] | [4.15, 5.1] | 10 | 61.0 | | [56, 64] | [58.5, 62.5] | 3 | 16.4 | | [12.6, 86] | [14.4, 44.4] | 11 |  |
| **75+** | 66.5 | [58, 75] | [62.3, 70.8] | 2 | 59.7 | [46.9, 118] | [53, 65.3] | 10 | 182.0 | | [174232] | [178, 207] | 3 | 183.8 | | [144.4, 337.4] | [167.1, 238.3] | 12 |  |
| **65+** | 87.0 | [46, 128] | [66.5, 107.5] | 2 | 50.0 | [49, 51] | [49.5, 50.5] | 2 | 91.25 | | [53.7, 124.5] | [72.5, 106.8] | 18 | 108.0 | | [52.7, 156] | [80.4, 132] | 3 |  |
| **all ages** | 47.0 | [47, 47] | [47, 47] | 1 | 78.0 | [15102] | [61.3, 87.5] | 8 | 53.3 | | [41.6, 77.4] | [49, 62.9] | 17 | 68.0 | | [27.2, 263] | [61.6, 196] | 21 |  |

**Note:** C&R = circulatory and respiratory; ARI = acute respiratory infection; P&I = Influenza and pneumonia; ALRI = acute lower respiratory infection. No. = the number of data-points used for calculating median, range and IQR. If studies reported estimates for multiple years or seasons, then this study contributes to multiple data points.

## Table S14. Median and range of reported estimates of ALRI, P&I, C&R and ARI hospitalisation associated with RSV per 100,000 by age group in high income countries when excluding low-quality studies.

|  | **RSV-ALRI hospitalisation** | | | | **RSV-P&I hospitalisation** | | | | **RSV-C&R hospitalisation** | | | | **RSV-ARI hospitalisation** | | | |
| --- | --- | --- | --- | --- | --- | --- | --- | --- | --- | --- | --- | --- | --- | --- | --- | --- |
| **Age group**  **(years)** | **median** | **Range** | **[P25, P75]** | **No.** | **median** | **range** | **[P25, P75]** | **No.** | **median** | **range** | **[P25, P75]** | **No.** | **median** | **range** | **[P25, P75]** | **No.** |
| **<1** | 3360 | [608.2, 13317.2] | [2975.0, 6783.4] | 11 | 157.5 | [54.0, 269.0] | [72.0, 186.4] | 13 | 2227.7 | [1817.2, 3150.7] | [2029.5, 2640.4] | 15 | 2109.3 | [1353.0, 4400.0] | [1743.8, 3407.5] | 18 |
| **1-4** | 94.0 | [34.6, 2366.0] | [77.0, 972.4] | 11 | 78.5 | [59.3, 117.0] | [69.4, 89.0] | 13 | 176.6 | [139.5, 260.4] | [158.4, 187.9] | 15 | 171 | [96.4, 4470.0] | [116.7, 514.0] | 21 |
| **5-17** | 1.8 | [1.8, 1.8] | [1.8, 1.8] | 1 | __ | __ | __ | __ | __ | __ | __ | __ | 1.6 | [0.5, 54.0] | [1.3, 13.0] | 14 |
| **18-49** | __ | __ | __ | __ | 6 | [2.0, 7.2] | [5.5, 6.6] | 9 | __ | __ | __ | __ | 11.2 | [1.2, 14.2] | [10.0, 12.8] | 11 |
| **50-64** | __ | __ | __ | __ | 6.3 | [5.1, 10.0] | [5.6, 6.7] | 9 | 12.35 | [7.3, 19.9] | [9.6, 14.9] | 16 | 26.4 | [5.0, 31.9] | [23.6, 28.7] | 11 |
| **65-74** | 12 | [12.0, 12.0] | [12.0, 12.0] | 1 | 4.5 | [3.7, 25.0] | [4.1, 4.8] | 9 | 62.5 | [61.0, 64.0] | [61.8, 63.2] | 2 | 16.05 | [12.6, 84.0] | [14.2, 17.5] | 10 |
| **75+** | 58 | [58.0, 58.0] | [58.0, 58.0] | 1 | 58.3 | [46.9, 118.0] | [52.4, 62.0] | 9 | 207 | [182.0, 232.0] | [194.5, 219.5] | 2 | 179.5 | [144.4, 337.4] | [165.2, 227.8] | 11 |
| **65+** | 128 | [128.0, 128.0] | [128.0, 128.0] | 1 | 51 | [51.0, 51.0] | [51.0, 51.0] | 1 | 89.5 | [53.7, 124.5] | [71.0, 106.0] | 17 | 80.35 | [52.7, 108.0] | [66.5, 94.2] | 2 |

**Note:** C&R = circulatory and respiratory; ARI = acute respiratory infection; P&I = Influenza and pneumonia; ALRI = acute lower respiratory infection. No. = the number of data-points used for calculating median, range and IQR. If studies reported estimates for multiple years or seasons, then this study contributes to multiple data points.

## Table S15. Median, IQR and range of reported estimates of all-cause, C&R, ARI and P&I mortality associated with RSV per 100,000 by age group globally (regardless of income level).

|  | **RSV all-cause mortality** | | | | | **RSV-P&I mortality** | | | | | **RSV-C&R mortality** | | | | **RSV-ARI mortality** | | | |
| --- | --- | --- | --- | --- | --- | --- | --- | --- | --- | --- | --- | --- | --- | --- | --- | --- | --- | --- |
| **Age group (years)** | **median** | **range** | **[P25, P75]** | **No.** | **median** | | **range** | **[P25, P75]** | **No.** | **median** | | **range** | **[P25, P75]** | **No.** | **median** | **range** | **[P25, P75]** | **No.** |
| **<1** | 8.4 | [5.4, 143.4] | [6, 15.1] | 5 | 16.8 | | [2.0, 33.0] | [2.8, 31.1] | 4 | 5.3 | | [5.3, 5.3] | [5.3, 5.3] | 1 | 19.0 | [2.48, 42] | [2.8, 36.8] | 4 |
| **1-4** | 0.9 | [0.01, 7] | [0.7, 2.4] | 4 | 1.6 | | [0.01, 3.3] | [0.08, 3.08] | 4 | 0.5 | | [0.2, 0.7] | [0.3, 0.6] | 2 | 3.0 | [0.3, 5] | [0.66, 4] | 5 |
| **5-17** | 0.3 | [0.1, 3.7] | [0.2, 1.2] | 4 | 0.1 | | [0.01, 0.6] | [0.01, 0.3] | 4 | __ | | __ | __ | __ | __ | __ | __ | __ |
| **18-49** | 0.3 | [0.01, 9.9] | [0.1, 2.4] | 5 | 0.6 | | [0.01, 1.4] | [0.2, 1.02] | 4 | 0.7 | | [0.4, 1] | [0.6, 0.9] | 3 | 1.2 | [1, 2.1] | [1.1, 1.65] | 3 |
| **50-64** | 6.0 | [1.9, 15.7] | [2.2, 7.8] | 5 | 1.6 | | [0.5, 4] | [0.7, 2.8] | 4 | 4.7 | | [4.0, 8.0] | [4.4, 6.4] | 3 | 5.3 | [4.6, 5.9] | [5.0, 5.6] | 2 |
| **65-74** | 10.0 | [8.7, 29] | [10, 10] | 11 | 5.1 | | [4.3, 17] | [4.7, 11.1] | 3 | __ | | __ | __ | __ | 4.2 | [4.2, 4.2] | [4.2, 4.2] | 1 |
| **75+** | 80.0 | [46.4, 155] | [70, 90] | 11 | 27.4 | | [15.7, 121] | [21.6, 74.2] | 3 | __ | | __ | __ | __ | 16.0 | [16, 16] | [16, 16] | 1 |
| **<5** | 11.0 | [0.8, 35.2] | [9.5, 11.5] | 15 | 9.0 | | [0.5, 10] | [8, 10] | 14 | __ | | __ | __ | __ | 11.4 | [0.9, 38.5] | [7.7, 19.2] | 4 |
| **65+** | 88.0 | [29.6, 622] | [61.2, 101.7] | 7 | 36.6 | | [7.2, 66] | [21.9, 51.3] | 2 | 68.1 | | [26.5, 113] | [47.3, 90.6] | 3 | __ | __ | __ | __ |
| **all ages** | 6.6 | [0.6, 17.1] | [5.1, 13.2] | 9.0 | 2.2 | | [1.0, 11.0] | [1.9, 4.4] | 4 | 6.0 | | [4.3, 20] | [5.15, 13] | 3 | 7.6 | [7.3, 7.8] | [7.4, 7.7] | 2 |

**Note:** C&R = circulatory and respiratory; ARI = acute respiratory infection; P&I = Influenza and pneumonia. No. = the number of data-points used for calculating median, range and IQR. If studies reported estimates for multiple years or seasons, then this study contributes to multiple data points.

## Table S16. Median, IQR and range of reported estimates of all-cause, C&R, ARI and P&I mortality associated with RSV per 100,000 by age group in high income countries.

|  | **RSV all-cause mortality** | | | | | **RSV-P&I mortality** | | | | | **RSV-C&R mortality** | | | | | **RSV-ARI mortality** | | | | |
| --- | --- | --- | --- | --- | --- | --- | --- | --- | --- | --- | --- | --- | --- | --- | --- | --- | --- | --- | --- | --- |
| **Age group (years)** | **median** | **range** | **[P25, P75]** | **No.** | **median** | | **range** | **[P25, P75]** | **No.** | **median** | | **range** | **[P25, P75]** | **No.** | **median** | | **range** | **[P25, P75]** | **No.** |  |
| **<1** | 7.2 | [5.4, 15.1] | [5.9, 10.1] | 4 | 2.6 | | [2.0, 3.1] | [2.3, 2.8] | 2 | 5.3 | | [5.3, 5.3] | [5.3, 5.3] | 1 | 2.7 | | [2.5, 2.9] | [2.6, 2.8] | 2 |  |
| **1-4** | 0.9 | [0.01, 0.9] | [0.5, 0.9] | 3 | 0.06 | | [0.01, 0.1] | [0.03, 0.08] | 2 | 0.5 | | [0.2, 0.7] | [0.3, 0.6] | 2 | 0.7 | | [0.3, 3.0] | [0.5, 1.8] | 3 |  |
| **5-17** | 0.2 | [0.1, 0.2] | [0.1, 0.2] | 2 | 0.01 | | [0.01, 0.01] | [0.01, 0.01] | 2 | __ | | __ | __ | __ | __ | | __ | __ | __ |  |
| **18-49** | 0.1 | [0.01, 0.3] | [0.06, 0.2] | 3 | 0.1 | | [0.01, 0.2] | [0.06, 0.2] | 2 | 0.7 | | [0.4, 1] | [0.6, 0.9] | 3 | 1.0 | | [1.0, 1.0] | [1.0, 1.0] | 1 |  |
| **50-64** | 4.1 | [1.9, 7.8] | [2.1, 6.5] | 4 | 0.8 | | [0.5, 4.0] | [0.7, 2.4] | 3 | 4.7 | | [4.0, 8.0] | [4.4, 6.4] | 3 | __ | | __ | __ | __ |  |
| **65-74** | 10.0 | [8.7, 29] | [10.0, 10.0] | 10 | 10.7 | | [4.3, 17.0] | [7.5, 13.8] | 2 | __ | | __ | __ | __ | __ | | __ | __ | __ |  |
| **75+** | 80.0 | [46.4, 155.0] | [70.0, 90.0] | 10 | 74.2 | | [27.4, 121.0] | [50.8, 97.6] | 2 | __ | | __ | __ | __ | __ | | __ | __ | __ |  |
| **65+** | 70.1 | [29.6, 622.0] | [46.5, 211.5] | 4 | 36.6 | | [7.2, 66] | [21.9, 51.3] | 2 | 68.1 | | [26.5, 113.0] | [47.3, 90.6] | 3 | __ | | __ | __ | __ |  |
| **all ages** | 5.3 | [0.6, 15.0] | [3.1, 8.7] | 4 | 2.2 | | [1.0, 11.0] | [1.6, 6.6] | 3 | 6.0 | | [4.3, 20] | [5.15, 13.0] | 3 | __ | | __ | __ | __ |  |

**Note:** C&R = circulatory and respiratory; ARI = acute respiratory infection; P&I = Influenza and pneumonia. No. = the number of data-points used for calculating median, range and IQR. If studies reported estimates for multiple years or seasons, then this study contributes to multiple data points.

## Table S17. Median, IQR and range of reported estimates of all-cause, C&R, ARI and P&I mortality associated with RSV per 100,000 by age group in upper-middle income countries.

|  | **RSV all-cause mortality** | | | | | **RSV-P&I mortality** | | | | | **RSV-ARI mortality** | | | | |
| --- | --- | --- | --- | --- | --- | --- | --- | --- | --- | --- | --- | --- | --- | --- | --- |
| **Age group (years)** | **median** | **range** | **[P25, P75]** | **No.** | **median** | | **range** | **[P25, P75]** | **No.** | **median** | | **range** | **[P25, P75]** | **No.** |  |
| **<1** | 143.4 | [143.4, 143.4] | [143.4, 143.4] | 1 | 31.7 | | [30.4, 33] | [31.1,32.4] | 2 | 38.5 | | [35, 42] | [36.8，40.3] | 2 |  |
| **1-4** | 7.0 | [7.0, 7.0] | [7.0, 7.0] | 1 | 3.2 | | [3.0, 3.3] | [3.1,3.2] | 2 | 4.5 | | [4.0, 5.0] | [4.3,4.8] | 2 |  |
| **5-17** | 2.1 | [0.4, 3.7] | [1.2,2.9] | 2 | 0.4 | | [0.2, 0.6] | [0.3,0.5] | 2 | __ | | __ | __ | __ |  |
| **18-49** | 0.3 | [0.01, 9.9] | [0.1,2.4] | 2 | 1.2 | | [0.9, 1.4] | [1.0,1.3] | 2 | 1.7 | | [1.2, 2.1] | [1.4,1.9] | 2 |  |
| **50-64** | 15.7 | [15.7, 15.7] | [15.7, 15.7] | 1 | 2.4 | | [2.4, 2.4] | [2.4, 2.4] | 1 | 5.3 | | [4.6, 5.9] | [4.9,5.6] | 2 |  |
| **65-74** | 22.0 | [22.0, 22.0] | [22.0, 22.0] | 1 | 5.1 | | [5.1, 5.1] | [5.1, 5.1] | 1 | 4.2 | | [4.2, 4.2] | [4.2,4.2] | 1 |  |
| **75+** | 81.7 | [81.7, 81.7] | [81.7, 81.7] | 1 | 15.7 | | [15.7, 15.7] | [15.7, 15.7] | 1 | 16.0 | | [16, 16] | [16,16] | 1 |  |
| **<5** | 11.0 | [7.0, 35.2] | [10.0,11.5] | 14 | 9.0 | | [6.0, 10] | [8.0,10.0] | 13 | 12.7 | | [0.4, 7.6] | [0.8,1.2] | 3 |  |
| **65+** | 92.9 | [70.2, 110.5] | [81.6,101.7] | 3 | __ | | __ | __ | __ | __ | | __ | __ | __ |  |
| **all ages** | 10.5 | [5.1, 17.1] | [6.6,13.2] | 5 | 2.2 | | [2.2, 2.2] | [2.2, 2.2] | 1 | 7.6 | | [7.3, 7.8] | [7.4,7.7] | 2 |  |

**Note:** ARI = acute respiratory infection; P&I = Influenza and pneumonia. No. = the number of data-points used for calculating median, range and IQR. If studies reported estimates for multiple years or seasons, then this study contributes to multiple data points.

## Table S18. Median, IQR and range of reported estimates of all-cause, C&R, ARI and P&I mortality associated with RSV per 100,000 by age group in high income countries when excluding low-quality studies.

|  | **RSV all-cause mortality** | | | | | **RSV-ARI mortality** | | | | |
| --- | --- | --- | --- | --- | --- | --- | --- | --- | --- | --- |
| **Age group (years)** | **median** | **range** | **[P25, P75]** | **No.** | **median** | | **range** | **[P25, P75]** | **No.** |  |
| **<1** | 11.8 | [8.4, 15.1] | [ 10.1, 13.4] | 2 | 2.7 | | [2.5, 2.9] | [2.6, 2.8] | 2 |  |
| **1-4** | 0.9 | [0.9, 0.9] | [0.9, 0.9] | 1 | 0.5 | | [0.3, 0.7] | [0.4, 0.6] | 2 |  |
| **5-17** | 0.2 | [0.2, 0.2] | [0.2, 0.2] | 1 | __ | | __ | __ | __ |  |
| **18-49** | 0.1 | [0.1, 0.1] | [0.1, 0.1] | 1 | __ | | __ | __ | __ |  |
| **50-64** | 1.9 | [1.9, 1.9] | [1.9, 1.9] | 1 | __ | | __ | __ | __ |  |
| **65-74** | 10.0 | [ 10.0, 10.0] | [ 10.0, 10.0] | 8 | __ | | __ | __ | __ |  |
| **75+** | 80.0 | [ 70.0, 90.0] | [ 70.0, 90.0] | 8 | __ | | __ | __ | __ |  |
| **65+** | 337.1 | [ 52.1, 622.0] | [194.6, 479.5] | 2 | __ | | __ | __ | __ |  |

**Note:** ARI = acute respiratory infection. No. = the number of data-points used for calculating median, range and IQR. If studies reported estimates for multiple years or seasons, then this study contributes to multiple data points.
